# The current state of artificial intelligence-based invasive coronary angiography image analysis: a systematic review

**DOI:** 10.1093/ehjdh/ztag110

**Published:** 2026-07-13

**Authors:** Viktor O van der Valk, Wouter P M van der Loo, Nico Bruining, Jouke Dijkstra, Douwe E Atsma, Marius Staring, Roderick W C Scherptong

**Affiliations:** Department of Radiology, Leiden University Medical Center, Albinusdreef 2, 2333 ZA Leiden, The Netherlands; Department of Cardiology, Leiden University Medical Center, Albinusdreef 2, 2333 ZA Leiden, The Netherlands; Thoraxcenter, Erasmus MC Rotterdam, Dr. Molenwaterplein 40, 3015 GD Rotterdam, The Netherlands; Department of Radiology, Leiden University Medical Center, Albinusdreef 2, 2333 ZA Leiden, The Netherlands; Department of Cardiology, Leiden University Medical Center, Albinusdreef 2, 2333 ZA Leiden, The Netherlands; Department of Radiology, Leiden University Medical Center, Albinusdreef 2, 2333 ZA Leiden, The Netherlands; Department of Cardiology, Leiden University Medical Center, Albinusdreef 2, 2333 ZA Leiden, The Netherlands

**Keywords:** Artificial intelligence, Invasive coronary angiography, Image analysis, Deep learning

## Abstract

Invasive coronary angiography (ICA) is the reference standard for diagnosing coronary artery disease and guiding percutaneous coronary intervention, yet clinical interpretation relies largely on visual assessment, which is variable and often requires additional invasive testing to assess functional significance. Artificial intelligence (AI)–based analysis of ICA images has emerged as a potential solution to automate interpretation, improve reproducibility, and extract anatomical and physiological information directly from angiograms. We conducted a systematic review of AI applications for ICA image analysis, registered in PROSPERO and reported according to PRISMA guidelines. A total of 134 studies were included, covering tasks across the ICA workflow, including automated frame selection, vessel segmentation, lesion detection and quantification, prediction of invasive physiological indices, coronary anatomy labelling, image registration and reconstruction, outcome prediction and left ventricular function estimation. Most studies focused on vessel segmentation and lesion assessment, generally demonstrating high internal performance but marked heterogeneity in datasets, reference standards, evaluation metrics, and validation strategies. While earlier work relied predominantly on single-centre retrospective validation, more recent studies increasingly incorporate multi-centre data, external validation and prospective evaluation. AI-based prediction of invasive physiological indices appears particularly promising for reducing reliance on wire-based measurements, though robust prospective evaluation remains limited. Overall, AI-based ICA analysis has progressed from technical feasibility studies towards clinically oriented applications. However, challenges in generalizability, methodological standardization, and workflow integration must be addressed to enable reliable clinical adoption.

## Graphical Abstract

**Graphical Abstract:**
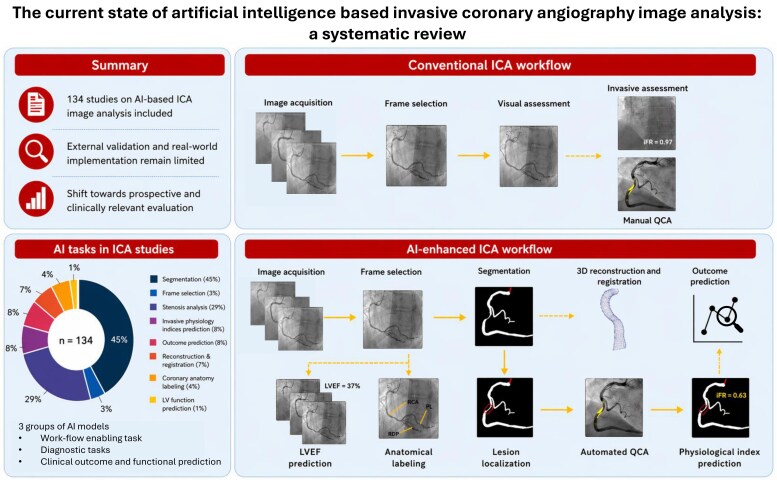

## Introduction

Invasive coronary angiography (ICA) is the gold standard for diagnosing coronary artery disease (CAD) and guiding percutaneous coronary intervention (PCI).^1^ Clinical decision-making during ICA largely relies on visual interpretation, a process prone to inter- and intra-observer variability and limited in its ability to detect conditions without obstructive lesions, such as coronary microvascular dysfunction (CMD). These limitations may lead to inappropriate interventions or missed therapeutic opportunities.^2–4^

Quantitative coronary angiography (QCA) provides a more robust assessment, but still depends on manual input, reducing efficiency and reproducibility.^2,5^ Additionally, it lacks physiological insight, often necessitating additional invasive procedures such as fractional flow reserve (FFR) and instantaneous wave-free ratio (iFR) measurements or intravascular imaging.^6,7^ While valuable, these techniques can increase cost, procedure time and complexity.^8^ In this context, artificial intelligence (AI) may add value by automating specific aspects of ICA and serving as a complementary tool alongside existing diagnostic methods. It could also help extract additional functional and anatomical information from standard angiograms.

Despite growing interest, AI development for ICA has progressed more slowly than in other imaging modalities, due to challenges such as dynamic contrast flow, vessel overlap, motion artefacts and limited availability of large, annotated datasets.^9^ Nevertheless, early research suggests that AI-based ICA analysis has the potential to support more comprehensive assessments and reduce reliance on additional testing.^10^ If successfully validated, such approaches may help streamline workflows and expand access to advanced diagnostic insights.

This review critically evaluates the current landscape of AI-based ICA analysis. It summarizes addressed clinical tasks, input data, model performance and validation levels, and identifies key gaps to guide future research and clinical translation.

## Methods

This systematic review was prospectively registered in PROSPERO (CRD420251027574).

The manuscript is reported in accordance with the Preferred Reporting Items for Systematic Reviews and Meta-Analyses (PRISMA) guidelines.^11^

### Search strategy and inclusion

Eligible studies were identified by systematically searching PubMed, EMBASE, Web of Science and IEEE Xplore. Additional studies were identified by screening the reference lists of included papers and a supplementary search on ArXiv. The final search was conducted on 1 April 2025. Detailed search strategies for each database are provided in Supplementary material online, A. Studies were included if the application of AI-based methods for the analysis of ICA images was investigated. Studies were excluded if they used only non-AI-based approaches, applied AI methods to non-image data only, were not published in English, or if no full-text paper was available. Furthermore, review articles, editorials, research letters, and animal studies were excluded. After removal of duplicates, titles and abstracts were independently screened by two reviewers. Discrepancies were resolved through discussion. Full texts of potentially eligible studies were then assessed for final inclusion.

### Study evaluation and data extraction

From each included study, the following data were extracted: Datasets used for training and validation, characteristics of the AI models, tasks addressed, performance metrics, and the level of model validation, ranging from model development to prospective evaluation. When multiple models or evaluation settings were reported for the same task, the author-defined primary or best-performing model was selected. If no primary model was specified, the model with the highest performance on a held-out test set was selected, with test-set results prioritized over validation-set results.

### Bias assessment

No formal risk of bias assessment tool was applied due to the absence of standardized tools applicable to both technical and clinical AI studies. Instead, the level of model validation (internal, external or prospective evaluation) and the use of multi-centre data were used as proxies for assessing model robustness and generalizability. Detailed information on datasets, model characteristics, and validation is provided in Supplementary material online, *Tables S1*–S16

## Results

### Study selection and characteristics

**Figure 1:**
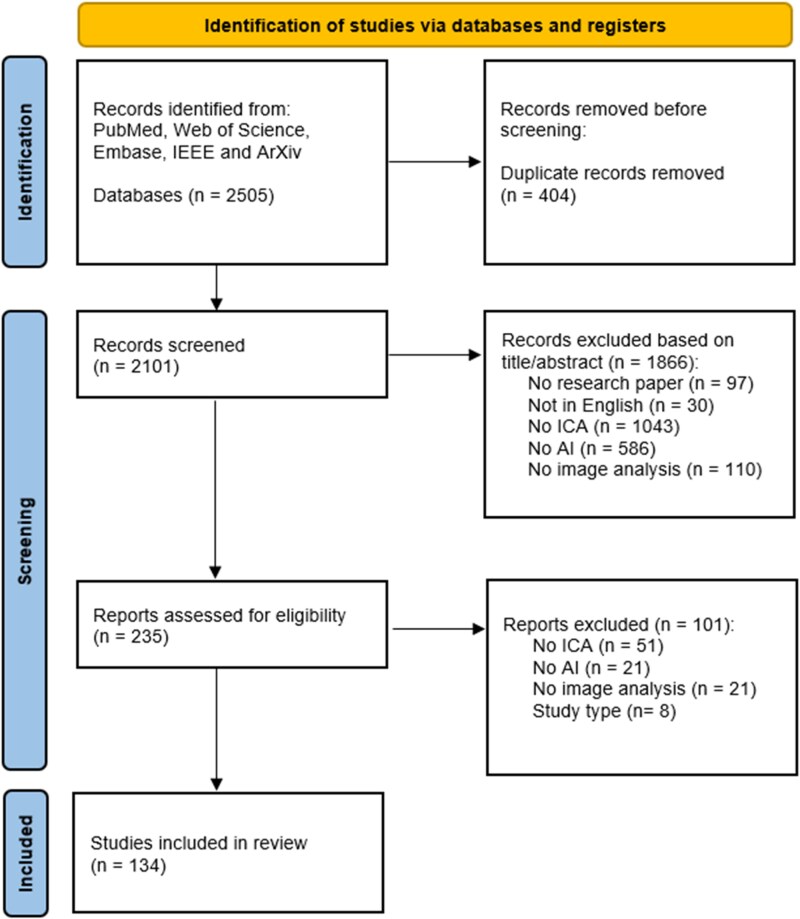
Study selection flowchart describing inclusion steps with numbers and reasons for exclusion.

**Figure 2:**
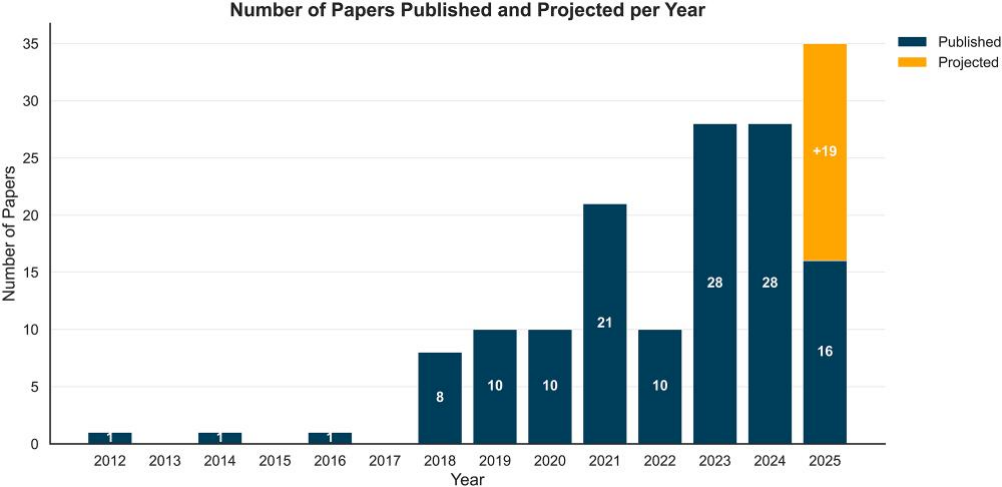
Number of papers published and projected per year. Projection for 2025 based on publication patterns in 2023 and 2024.

The search identified 2505 articles. After de-duplication, 2101 titles/abstracts were screened and 1866 excluded. Of 235 full-text articles, 101 were excluded (no ICA data (*n* = 51), non-AI methods (*n* = 21), no direct image analysis (*n* = 21), ineligible study type (*n* = 8)), leaving 134 included studies (*Figure 1*). An overview is provided in Supplementary material online, B. Research activity increased after 2018 (*Figure 2*).

**Figure 3:**
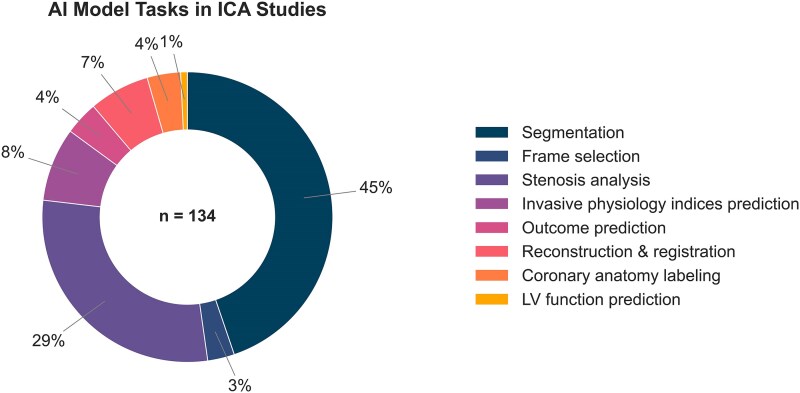
Distribution of model tasks across included studies.

Studied tasks included vessel segmentation (*n* = 60, 45%), lesion assessment (*n* = 39, 29%), invasive physiology indices prediction (*n* = 11, 8%), reconstruction/registration (*n* = 9, 7%), outcome prediction (*n* = 5, 4%), coronary anatomy labelling (*n* = 5, 4%), frame selection (*n* = 4, 3%), and left ventricular (LV) function prediction (*n* = 1, 1%) (*Figure 3*). Segmentation and anatomical identification frequently served both as standalone objectives and as components within multi-stage pipelines.

**Figure 4:**
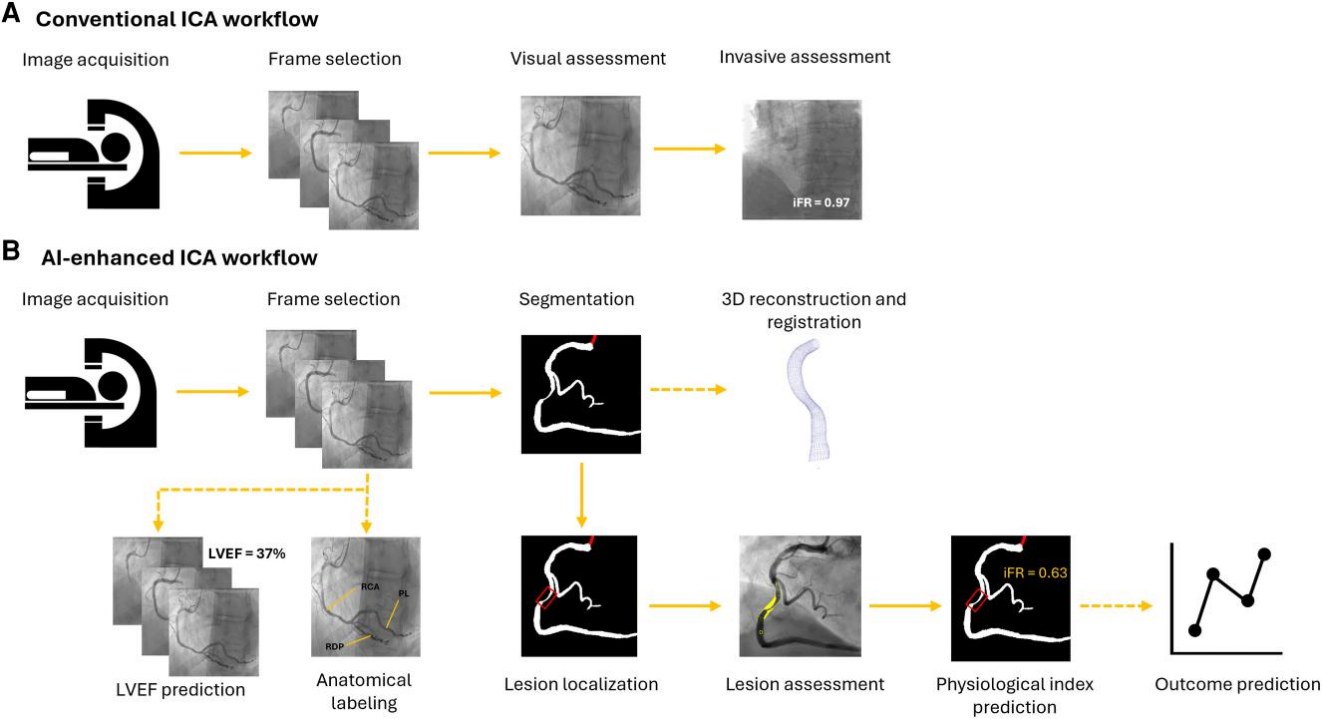
Conventional and AI-enabled workflows for coronary angiography analysis. The upper panel illustrates the standard workflow for visual and physiological interpretation of invasive coronary angiography. The lower panel shows an AI-enabled approach that automates key analytical steps supporting faster, standardized, and quantitative assessment.

For synthesis, each study was assigned to a single primary task category, with detailed information on datasets, model characteristics, and validation provided in Supplementary material online, *Tables S1*–S16. Results are organized into three broader domains: workflow-enabling tasks, diagnostic assessment tasks, and clinical outcome and functional prediction, aligned with the ICA workflow illustrated in *Figure 4*. Because task definitions, reference standards, validation strategies, and reported metrics differed substantially, performance estimates were summarized descriptively and should not be interpreted as direct comparisons between task domains or as rankings of model performance.

### Workflow-enabling tasks

AI applications aimed at enabling the ICA workflow included frame selection, vessel segmentation, anatomical labelling, and image registration or reconstruction. These tasks support downstream diagnostic interpretation by standardizing inputs, improving visualization and enabling quantitative analysis.

Frame selection was addressed in four studies and represents an important component of ICA automation. A model was trained to asses diagnostic-quality of images, reaching an area under the ROC curve (AUC) of 0.95 in external validation. Other models addressed end-diastolic or contrast-related key-frame detection, reporting high accuracy or low frame-level error against ECG-based or manual reference standards. Dataset sizes varied widely, from 100 to 30 000 ICA sequences. Ground truths were predominantly based on manual annotation, except for the EDF detection model, which used ECG-based labels. Beyond standalone models, frame selection was frequently embedded within broader pipelines, e.g. through rule-based selection of frames with maximal vascular area determined by segmentation.^12^ Other approaches incorporated temporal constraints or attention mechanisms to focus on contrast-rich or anatomically informative frames, which improved downstream model performance.^13^

Vessel segmentation was the most extensively studied workflow-enabling task (*n* = 60, 45%) and forms the basis for downstream analyses such as stenosis assessment, anatomical labelling and QCA. The primary goal was to delineate the coronary lumen, either the full coronary tree (*n* = 53) or major arteries (LAD, LCx, or RCA, *n* = 7). Deep learning (DL) was the predominant approach (*n* = 57). CNN-based architectures predominated, with fewer studies using attention mechanisms, GANs, transformers, or semi-/unsupervised approaches to reduce annotation dependence. Most models (52/60) used single-frame inputs, while 8 employed full ICA sequences. Training datasets ranged widely, from 10 to 5941 frames for supervised single-frame models and from 24 to 255 sequences for supervised multi-frame models. Performance was generally high in internal validation across standard classification and overlap metrics. Accuracy ranged from 0.887 to 0.998, sensitivity from 0.602 to 0.999, NPV from 0.906 to 0.990, DSC from 0.659 to 0.998, AUC from 0.929 to 0.994, precision from 0.701 to 0.975 and IOU from 0.811 to 0.926. Eight studies performed external validation. Across these, model performance typically declined modestly when evaluated on external datasets (Yang *et al*., ^14^: F1 from 0.917 to 0.896; Park *et al*.,^15^: DSC d from 0.93 to 0.90), whereas some models exhibited more pronounced reductions (Shi *et al*.,^16^: F1 from 0.82 to 0.736). Models demonstrating smaller performance drops during external validation were generally trained on larger image datasets (see Supplementary material online, *Tables S3*–S4). One study directly evaluated clinical impact, reporting that AI-based vessel contours significantly improved the accuracy of visual stenosis estimation compared to QCA (agreement 60.4% vs. 30.1%, *P* < 0.001).^17^

Anatomical labelling was less frequently studied (*n* = 5) but represents an important step in structuring for downstream analysis. One model for LCA/RCA identification was developed, achieving an average accuracy of 0.997. Two studies extended anatomical labelling to a finer granularity, using graph neural networks (GNNs) to label coronary artery segments (LM, LAD, LCx, marginal obtuse and diagonal branches). Zhao *et al*.,^18^ reported an accuracy of 0.921, precision 0.926, recall 0.926, and F1-score 0.926, trained on multi-centre data. Zhao *et al*.^18^ used multi-centre data with site-based external evaluation, which revealed reduced performance, highlighting limited generalizability. Two studies addressed anatomical features beyond vessel labelling. Cobo *et al*. (2023) developed a model for coronary tortuosity detection with an accuracy of 0.870, matching expert consensus. Hatfaludi *et al*. (2023) developed an algorithm detecting collateral circulation, achieving an accuracy of 0.795. All studies, except Zhao *et al*.,^18^ used retrospective, single-centre datasets without external validation.

Reconstruction and registration were investigated in nine studies (registration *n* = 5; reconstruction or enabling steps *n* = 4), focusing on improving geometric accuracy, motion correction, and multi-modal integration. All were model development studies; two included external validation. Registration refers to aligning images, either between ICA frames or across modalities. Reconstruction involves deriving 3D coronary anatomy from 2D projections, aiming to improve motion correction, geometric accuracy, and multi-modal integration for quantitative analysis.

Three image-pair registration models were developed. Kim *et al*.^19^ developed a CNN-GNN model for aligning ICA frame pairs, validated externally. With similar internal and external performance (accuracy 0.730 and 0.732, respectively). Kim *et al*.^19^ created a model for anatomical landmark pairing, as basis for real-time dynamic coronary road mapping, achieving an accuracy of 1.04 mm. Fan *et al*. (2019) developed a CNN-based model for image pair registration, reporting a root-mean-square error of 9.79 pixels. Other registration tasks targeted sequence-level or multi-modal alignment. Royer *et al*. (2020) aligned ICA sequences to the cardiac cycle using ECG-based ground truth to facilitate 3D reconstruction. Yan *et al*. (2023) registered 3D CCTA centrelines to 2D ICA images using a CNN with reinforcement learning, reporting a mean projection error of 2.52 pixels.

The remaining studies focused on reconstruction or enabling tasks. Bransby *et al*. (2023) reconstructed 3D coronary meshes from two ICA projections by using a combination of a CNN and a GNN, achieving a mean absolute vessel geometry error of 0.35 mm. Li *et al*. (2025) fused ICA frames and OCT pullbacks using a transformer-based model, achieving near-expert registration accuracy. Dinescu *et al*. (2023) enhanced ICA image quality on synthetically degraded images. Fang *et al*. (2020) developed a model to estimate respiratory motion signals directly from ICA sequences, trained on manual diaphragm motion tracking, achieving high correlation with reference signals (*r* = 0.92–0.95). Overall, these approaches demonstrate diverse strategies to support motion correction, multi-modal alignment and 3D reconstruction, although most remain at the model development stage with limited external validation.

### Diagnostic assessment

AI applications for diagnostic assessment focus primarily on lesion characterization and functional significance. Lesion assessment was the most extensively studied diagnostic task (*n* = 39), encompassing lesion localization, stenosis quantification and lesion type classification. Thirty eight studies used DL models. Inputs were single-frame in 25 studies, multi-frame in 11 studies, and patch-based in 3 studies. Fourteen algorithms targeted only specific segments of the coronary artery tree. One study developed dedicated models for both RCA and LCA.^20^ The remaining studies assessed both jointly.

Methodologically, approaches varied substantially. Approaches included bounding-box localization, segmentation-based analysis, classification without explicit localization, direct stenosis prediction, and automated QCA. Reported tasks were binary stenosis detection, multiclass severity grading, and continuous prediction of diameter reduction. Six studies additionally classified lesion types (e.g. CTO, bifurcation, thrombus, dissection, calcification). Datasets and reference standards were highly heterogeneous, limiting direct comparability across studies. Datasets ranged from 50–84 011 images and 98–93 326 sequences. Four studies included synthetic data alongside real-world images. Ground truth varied: Visual assessment (*n* = 22), QCA (*n* = 9), IVUS (*n* = 1), OCT (*n* = 1), or unspecified (*n* = 4). This heterogeneity extended to stenosis grading systems. There was substantial variability in the reported metrics. Accuracy ranged from 0.600 to 0.940, AUC 0.450 to 0.981, sensitivity 0.511 to 0.990, precision 0.220 to 0.990, F1-score 0.314 to 0.980, specificity 0.600 to 0.983, and NPV 0.869 to 0.924.

External validation was limited (*n* = 5) and demonstrated variable generalizability, often influenced by differences in labelling and included cases. Moon *et al*.^12^ reported a minor reduction in AUC from 0.971 to 0.956 (external). Similarly, Labreque-Langlais *et al*.^21^ reported stable performance on an external cohort. Avram *et al*.^22^ demonstrated good generalization (AUC 0.869 external vs. 0.862 internal) but noted performance decline (AUC 0.775) when retrained on QCA labels, highlighting label inconsistencies. Eschen *et al*.^20^ observed a more substantial AUC drop (0.903–0.780 against FFR-based labels), partially attributed to a higher prevalence of intermediate lesions in the external dataset. Several studies evaluated commercially available AI-QCA software. Four studies evaluated the MPXA-2000 software (Medipixel). Moon *et al*.^23^ described its architecture of three integrated DL networks, reporting strong segmentation and vessel classification performance. Validation against IVUS showed moderate-to-strong correlations for anatomical parameters, though weaker correlations for stenosis metrics due to modality mismatches. Kim *et al*.^19^ demonstrated superior consistency of AI-QCA over visual assessment, closely aligned with manual QCA. Chae *et al*. (2025) reported a high lesion detection sensitivity of 93% and similarly strong correlations with manual QCA. The prospective FLASH trial (Kim Y *et al*.^10^) showed non-inferiority of AI-QCA-guided PCI compared to OCT guidance regarding minimal stent area, despite slightly higher rates of stent malposition.

Prediction of invasive physiological indices was investigated in 11 studies, representing a key extension from anatomical to functional assessment. Nine studies focused on FFR/iFR from ICA images and two explored related applications (Coronary flow reserve (CFR) from ICA images and CTO collateral physiology). Among FFR/iFR studies, four predicted FFR, three predicted iFR, and two both. Approaches varied in both prediction targets and model design. Models either classified lesions as haemodynamically significant (FFR ≤0.80 or iFR ≤0.89, *n* = 3) or predicted continuous physiological values (*n* = 6). Five studies focused on major coronary arteries only, one on the LAD only, and the remaining three on the whole coronary vascular tree. Architectures were mainly CNN-based, with some transformer or attention-based variants. Inputs ranged from single frames to multi-image approaches; one study incorporated clinical variables, although image-only models performed best. Ground truth was primarily based on wire-based FFR or iFR, except for Omori *et al*.^24^ and Zhang *et al*., which used computational fluid dynamics-based synthetic data for training and real-world data for validation. Dataset sizes ranged widely, from 31 to 1500 patients and up to 27 000 synthetic cases.

Reported performance was variable but generally promising, particularly for classification tasks.

AUC values ranged from 0.800 to 0.950, with accuracies up to 0.93, while for continuous predictions, mean absolute errors ranged from 0.005 to 0.05. Beyond FFR/iFR, Zhao *et al*.^18^ created a model for CFR prediction, combining vessel segmentation with frame-wise velocity estimation. CFR was successfully computed in 91.3% and correlated moderately with the TIMI frame count (*r* = 0.51). Liu *et al*. (2023) used a model for quantifying collateral physiology in CTOs, calculating collateral flow indices and tracking changes before and after PCI. The model’s outputs aligned with Rentrop and CC collateral grading systems and detected changes post-revascularization.

All studies were retrospective, with limited external validation (*n* = 4), and heterogeneous validation strategies. Three studies were retrospective external validation studies, without model training.^24–26^ Ben-Assa *et al*.^25^ demonstrated strong external performance, without reported internal performance. Omori *et al*.^24^ and Zhang *et al*. (2024) validated on real-world ICA datasets following training on synthetic data. Zhang *et al*. (2024) described consistent performance across internal and external datasets; Omori *et al*.^24^ did not report performance on the internal dataset. De Filippo *et al*. (2024) conducted internal validation on a multi-centre dataset, while the remaining studies used single-centre data.

### Clinical outcome and functional prediction

Beyond diagnostic assessment, several AI applications predicted patient-level outcomes, procedural success, and cardiac functional parameters. Five studies investigated clinical outcome prediction: four focused on adverse cardiovascular events and one on CTO revascularization success. All models used ICA images as input, combined with clinical variables in three studies. Image-only approaches typically used patch-based per-stenosis representations, while others incorporated multi-view inputs to capture lesion characteristics. Ground truths were derived from electronic health records.

Prediction targets and modelling strategies varied substantially. Three models estimated, at lesion level, the risk of ACS within 5 years, while one predicted a composite endpoint of cardiac death, myocardial infarction and revascularization within 2 years. Most used CNNs (*n* = 4), with one study combining CNNs with an ANN and another using a GNN. Training datasets ranged from 90 to 451 patients and up to 671 patches. Model performance varied considerably, reflecting differences in dataset size, event rates and endpoint definitions. No study performed external validation. Two studies (Sun *et al*., 2024; Mahendiran *et al*.,^27^) used multi-centre trial data for training, whereas the remainder relied on single-centre cohorts. Reported AUCs for ACS prediction ranged from 0.627 to 0.810, with F1-scores between 0.167 and 0.875. Mahendiran *et al*.^27^ demonstrated improved performance compared with conventional angiographic predictors such as stenosis diameter, stenosis area, and QFR, while other studies showed more modest or comparable results. For CTO procedural success prediction, AI models did not outperform the established J-CTO score.

Functional prediction from ICA remains largely unexplored. One study, Avram *et al*.,^22^ developed a model to estimate LV ejection fraction (LVEF) from LCA ICAs, using echocardiography as ground truth. The model achieved an AUC of 0.911 for detecting LVEF<40%, with sensitivity 0.839 and specificity 0.813, consistent across internal and external cohorts.

Overall, AI-based prediction of clinical outcomes and cardiac function from ICA remains at an early stage, characterized by limited validation, heterogeneous endpoints, and modest performance, with current evidence insufficient to support routine clinical implementation.

### Methodological quality, validation, and potential bias across studies

Most studies were retrospective model development studies with internal validation only. External validation was limited, mainly in vessel segmentation (8/60), lesion assessment (5/39), and invasive physiological prediction (5/11), with isolated validation in other domains and none in outcome prediction. Prospective evaluation was rare, largely confined to AI-QCA, with one additional clinical evaluation in vessel segmentation. Despite some use of multi-centre data, external validation or prospective evaluation, evidence supporting generalizability remained limited, and varied across tasks.

Recurrent sources of potential bias were identified across multiple domains, including dataset composition, reference standards, annotation procedures, input selection, data splitting, validation strategy, metric reporting, and reporting completeness (*Table 1*). Dataset-related limitations included small or selected cohorts, retrospective single-centre data, and inconsistent reporting of patient characteristics, which may limit applicability to routine catheterization laboratory practice. Ground truth varied substantially across tasks, ranging from subjective visual assessment to physiological indices, and annotation procedures were inconsistently reported, potentially introducing label noise and limiting reproducibility. Data splitting was not always described sufficiently to confirm separation at patient, lesion, or acquisition level, introducing potential data leakage. Dependence on manually selected frames, preselected projections, cropped regions of interest, prior segmentation steps or synthetic data may further limit translation to real-world use. Finally, evaluation metrics were not standardized and were highly dependent on task definition, dataset composition, reference standard, and validation level. Direct numerical comparison across studies should therefore be interpreted cautiously. Few studies assessed uncertainty or performance near clinically relevant decision thresholds, although such information is essential for determining whether model outputs can support clinical decision-making.

**Table 1:** Structured overview of recurrent methodological limitations and potential sources of bias across included studies

Domain	Recurrent finding across studies	Potential source of bias	Implication for interpretation
**Dataset representativeness**	Datasets often used selected cohorts with variable case mix and acquisition settings	Limited representation of real-world ICA variability	Generalizability to routine ICA and different settings remains uncertain
**Dataset size**	Dataset size varied widely; many studies used relatively small test cohorts	Overfitting and unstable performance estimates	Performance should be interpreted in relation to the amount of data used
**Unit of analysis**	Data were variably reported at patient, lesion, vessel, sequence, frame, or pixel level	Sample size may appear inflated when multiple samples originate from the same patient	Reported performance may overstate patient-level robustness
**Reference standard**	Ground truth varied substantially across tasks and studies	Reference-standard variability and use of non-interchangeable labels	Metrics depend strongly on the selected target and cannot be directly compared across studies
**Annotation procedure**	Annotator expertise, number of annotators, consensus procedures and interobserver variability were inconsistently reported	Label noise, subjective annotation and limited reproducibility	Model performance may reflect agreement with a single annotator rather than a reproducible reference standard
**Image-quality handling**	Exclusion of poor contrast, motion artefacts, vessel overlap, foreshortening and small distal vessels was inconsistently described	Selection of technically favourable angiograms	Real-world performance may be lower than reported in curated datasets
**Input selection and preprocessing**	Some models depended on manual input or other preprocessing steps	Workflow-dependent performance and limited automation	Performance may not translate to fully automated clinical use
**Data splitting**	Separation of training and test data at patient, lesion or acquisition level not always clearly described	Data leakage between training and testing	Internal validation may overestimate performance
**Validation strategy**	Internal validation predominated; external and prospective evaluation were limited	Limited assessment of transportability and clinical robustness	Most models require further independent and prospective validation
**Metric reporting**	Reported metrics varied substantially across task domains	Inconsistent performance interpretation across studies	Should not be interpreted as direct model ranking
**Calibration and thresholds**	Calibration, uncertainty, and performance near clinically relevant thresholds were rarely assessed	Poorly characterized decision-boundary performance	Clinical usefulness may be uncertain despite high discrimination metrics
**Reporting completeness**	Dataset composition, preprocessing, architecture, training, code availability, and evaluation protocols were variably reported	Limited reproducibility and independent verification	Standardized reporting is needed for reliable validation and translation

Overall, current evidence is technically promising but methodologically heterogeneous. High reported performance should be interpreted in the context of dataset representativeness, annotation quality, input selection and validation design. For most models, evidence of robustness in independent, clinically representative and preferably prospective multi-centre cohorts remains limited.

## Discussion

This systematic review summarizes AI models for ICA image analysis, a rapidly growing field dominated by vessel segmentation and lesion assessment. CNNs predominated, with inputs ranging from single frames to ICA sequences and reference standards varying from visual assessment to invasive physiology. Although recent studies increasingly use multi-centre data and external validation, most remain retrospective model-development studies. High internal performance should therefore not be interpreted as clinical readiness. Overall, meaningful technical progress is evident, but methodological standardization, transparent reporting and external validation, prospective clinical evaluation, and workflow-based assessment remain essential for clinical integration.

### AI for workflow-enabling tasks

A substantial portion of the literature focuses on AI applications that support, rather than replace, conventional ICA interpretation by automating key preparatory steps such as frame selection, vessel segmentation, anatomical labelling and image registration or reconstruction. Despite differing technical objectives, these tasks share a common goal: improving input standardization, reducing operator dependency and enabling downstream quantitative analysis.

Appropriate frame selection improves input consistency, reduces computational burden, and supports real-time applications, such as QCA, where peak contrast opacification and clear vessel delineation are essential.^21,28^ Clinically, inconsistent frame choice contributes to inter-observer variability.^29^ Despite this relevance, relatively few studies have addressed frame selection, and most approaches are embedded within broader analysis pipelines. These typically rely on criteria such as maximal vascular area or temporal attention mechanisms to identify contrast-rich and anatomically informative phases.^12,30^

Vessel segmentation represents the most extensively studied workflow-enabling task and forms the technical backbone for many applications, including lesion quantification, anatomical labelling, and automated QCA. Across a large but methodologically heterogeneous literature base, segmentation models generally achieve high performance on major epicardial vessels. However, performance tends to decline in smaller or distal branches,^9,17^ which are clinically relevant in diffuse or complex coronary disease. This limitation likely reflects both anatomical complexity and the underrepresentation of small vessels in annotated datasets, highlighting a structural source of bias. Most studies used CNN-based models, while more recent approaches have explored attention mechanisms and transformers to improve temporal consistency and reduce annotation requirements. Semi-supervised and unsupervised strategies are promising in this regard but remain less extensively validated and are rarely compared directly with state-of-the-art supervised models.^31,32^ Importantly, although internal performance is often strong, generalizability remains variable. Studies based on larger and more heterogeneous datasets tended to show more stable external performance, underscoring dataset diversity as a key determinant of robustness.^14–16,33,34^ Clinically, accurate vessel segmentation can reduce inter-operator variability and enable automated QCA.^17,23^

Anatomical labelling extends segmentation by assigning meaning to identified vessels, further standardizing inputs for downstream tasks.^20,21^ While recent graph-based models better capture coronary relationships, current approaches remain limited to proximal segments.^18,35^ AI-based characterization of anatomical features such as tortuosity may provide additional clinical value. Tortuosity is associated with adverse outcomes and is inconsistently reported in routine practice, suggesting a potential role for automated and standardized assessment.^36,37^ In contrast, automated collateral detection likely adds limited clinical value beyond structured reporting. As with other tasks, limited validation and small, single-centre datasets constrain clinical applicability.

Image registration and reconstruction are the most technically ambitious applications. Their principal value lies in facilitating procedural guidance, especially when preprocedural CT or intravascular imaging is unavailable. In theory, ICA-based reconstruction could support complex interventions such as bifurcation PCI or CTO revascularization by providing a more structured anatomical map during the procedure. This concept parallels the broader movement towards image-guided coronary intervention, including CCTA-guided planning,^38^ but offers the practical advantage of being available during unplanned procedures. Although technically feasible, these methods remain preclinical, with limited evidence of robustness under real-world conditions. Their key challenge is no longer feasibility, but demonstrating added clinical value beyond established imaging modalities.

Overall, workflow-enabling applications are technically advanced but heterogeneous in maturity. Segmentation shows the strongest evidence base, whereas other tasks remain early-stage. Across all domains, limited dataset diversity and reliance on retrospective validation constrain generalizability. Future work should prioritize prospective evaluation and demonstrate real-world clinical benefit in catheterization laboratory workflows.

### AI for diagnostic assessment

AI for diagnostic assessment aims to move from anatomical description towards actionable decision-making by integrating lesion characterization with functional evaluation. Current approaches include AI-QCA and prediction of invasive physiological indices such as FFR and iFR.

Lesion assessment is the most extensively studied diagnostic application. Most models rely on single-frame inputs, reflecting conventional QCA workflows, neglecting temporal information that underpins clinical interpretation. Multi-frame models address this by incorporating contrast dynamics and vessel geometry, resulting in improved consistency and reduced false-positive findings.^21,30,39^ Bounding-box approaches are most common due to lower annotation requirements, but lack the precision needed for quantitative assessment. In contrast, segmentation-based models enable direct computation of QCA metrics^19^ and more detailed vessel characterization, aligning more closely with clinical decision-making, where the key question is not lesion presence but the need for revascularization.^19^ Despite encouraging technical performance, several methodological factors limit comparability and generalizability. Label variability is a major source of bias: models trained on visual assessment often perform less well when evaluated against QCA or FFR.^20,36^ While visual grading reflects routine clinical practice, its subjectivity reduces reproducibility. More objective reference standards, such as QCA or invasive physiology, improve model robustness but are less consistently available. In addition, dataset composition strongly influences performance. Models trained on large, multi-centre datasets generally demonstrate more stable external results,^22,23^ whereas performance declines are observed in cohorts enriched with intermediate lesions, which are both common and diagnostically challenging.^20,40^ This subgroup is particularly relevant, as it represents the clinical scenario in which AI could most meaningfully support decision-making. Among lesion assessment approaches, AI-based QCA represents the most advanced stage of clinical translation. A single-frame AI-QCA system has demonstrated non-inferiority to OCT-guided PCI in an RCT, supporting its potential clinical applicability.^10^ Most other approaches, particularly multi-view models, remain preclinical, with limited external validation.

AI-based prediction of invasive physiological indices extends ICA analysis from anatomical to functional assessment. The most mature applications focus on FFR and iFR, addressing their underuse in clinical practice.^8^ Across studies, models demonstrate promising performance, particularly in ruling out haemodynamically insignificant lesions, with high negative predictive values reported in external evaluations.^25,26,40^ This suggests potential utility as a gatekeeping tool to reduce unnecessary invasive measurements. Conversely, prediction uncertainty may help identify lesions requiring confirmatory testing. A notable strength of several models is their potential compatibility with clinical workflows. The model by Ben Assa *et al*.^25^ achieved high diagnostic accuracy without strict imaging requirements, generating rapid results with minimal manual input, key prerequisites for real-time procedural integration. However, these findings should be interpreted with caution, as the model was evaluated retrospectively and relied on core-laboratory analysis rather than real-time use by catheterization laboratory staff, limiting generalizability to routine clinical practice. Validation levels remain a key limitation. Most studies are retrospective, with few multi-centre or prospective evaluations. Some models rely on synthetic training data, which improves scalability but risks domain shift when applied to real-world datasets, particularly when internal validation is incompletely reported.^24^ Furthermore, precision near clinical decision thresholds remains critical, as small prediction errors may influence revascularization decisions.^41^ Beyond FFR and iFR, AI-based estimation of other physiological parameters remains exploratory. Models for CFR estimation and collateral flow quantification demonstrate technical feasibility but are typically trained on surrogate or non-invasive reference standards, lacking validation against invasive gold standards.^42^ As a result, their current clinical applicability is limited.

Importantly, a small number of systems have progressed beyond experimental validation to early clinical deployment. The AI-QCA platforms by Medipixel have received regulatory clearance, including FDA 510(k) clearance in the USA and approval in South Korea, while AutoCathFFR (MedHub Ltd.) has obtained regulatory approval in Japan.^43,44^ These examples highlight the feasibility of integrating AI-based analysis into clinical workflows, although real-world adoption remains limited. For most models, evidence is still restricted to retrospective or controlled settings, and real-time implementation in catheterization laboratories remains insufficiently evaluated. Future work should prioritize prospective multi-centre validation to enable reliable clinical integration.

### Clinical outcome and functional prediction

AI-based prediction of clinical outcomes and cardiac function from ICA remains at an early stage, with limited evidence and variable performance. These models address more complex, patient-level endpoints that depend on multiple clinical and biological factors not fully captured by angiographic imaging alone.

For outcome prediction, current models demonstrate moderate performance and, in some cases, modest improvements over simple angiographic metrics such as diameter stenosis or QFR. Predictive performance is limited, not outperforming established clinical risk models, reflecting the multifactorial nature of cardiovascular risk and the inherent constraints of image-based input alone.^27,45^ Prediction of procedural outcomes, such as CTO revascularization success, may represent a more practical near-term application, as these endpoints are more directly related to anatomical complexity and technical factors visible in ICA.^46^ However, evidence remains limited and current models have not demonstrated clear superiority over established scoring systems. Functional prediction from ICA has been explored only minimally. A single study demonstrated accurate estimation of reduced LVEF (<40%) from ICA sequences, but clinical utility of this approach appears limited. In acute settings, treatment decisions are driven by the need for immediate reperfusion irrespective of ventricular function, while in elective practice, echocardiography remains the standard modality for functional assessment.^1,47^

Overall, AI applications targeting outcome and functional prediction are at an early stage of development, characterized by small, predominantly single-centre datasets, heterogeneous endpoints and limited validation. Reported performance should therefore be interpreted with caution. Future progress will depend on larger, multi-centre datasets and multimodal approaches integrating imaging, clinical, and procedural data to better capture the complexity of cardiovascular risk and enable clinically meaningful prediction.

### Future directions

AI for ICA analysis has evolved from technology-driven proof-of-concept studies to models increasingly evaluated on larger, multi-centre datasets with external validation and prospective evaluation. The first RCT further reflects this maturation. Newer systems are designed around the ICA workflow, emphasizing minimal manual input and real-time compatibility. Clinical deployment will require interoperability with vendor-specific systems, acquisition protocols and reporting platforms, standardized ground-truth definitions aligned where possible with common data models, and minimal workflow burden. Prospective multicenter evaluation remains essential to assess accuracy, reliability, safety and workflow impact. Clinically, AI offers benefits beyond accuracy: reducing unnecessary invasive testing, improving safety and efficiency, and identifying uncertainty that may warrant further evaluation. It can also standardize interpretation, particularly valuable in resource-limited settings where adjunctive imaging is less accessible.^48^ Automation of anatomical labelling, quantitative analysis and structured reporting may further reduce clinician workload. Clinician–engineer collaboration remains essential to ensure models are both robust and practical. Large language models may aid dataset curation,^49^ while automated heart-team systems could combine imaging and clinical data to support evidence-based treatment recommendations.^50^ Standardized benchmarks and reporting frameworks remain essential for reliable cross-study comparison and clinical translation.

## Conclusion

AI-based ICA analysis has advanced rapidly, from technical feasibility studies to clinically oriented models evaluated on larger, externally validated datasets. Applications now span vessel segmentation, lesion assessment, physiology prediction, and outcome modelling. The main near-term value lies in reducing reliance on invasive adjunctive testing, standardizing interpretation, and improving procedural efficiency. Key challenges persist in generalizability, workflow integration, and standardized evaluation. Continued collaboration between clinicians and technical experts will be crucial to ensure clinical relevance. With sustained progress, AI-driven ICA could enhance diagnostic accuracy, streamline workflows and improve patient outcomes.

## Supplementary Material

ztag110_Supplementary_Data

## Data Availability

The data underlying this article are available in the article and in its online supplementary material.
