# Supplementary material for: The current state of artificial intelligence-based invasive coronary angiography image analysis: a systematic review
Source: Eur Heart J Digit Health. 2026 Jul 13;7(6):ztag110. doi: 10.1093/ehjdh/ztag110 (PMC13387071; doi:10.1093/ehjdh/ztag110)
Supplement: ztag110_Supplementary_Data [file ztag110_supplementary_data.docx]

**Supplement A. Search strategies**

PubMed

(”Coronary Angiography”[Majr] OR ”Coronary Angiography”[ti] OR ”Coronary Angiographies”[ti] OR ”coronary angiogram*”[ti]) AND (”Artificial Intelligence”[Majr] OR ”Artificial Intelligence”[ti] OR ”AI”[tiab] OR ”Image Processing, Computer-Assisted”[Majr] OR ”automatic image analysis”[ti] OR ”machine learning”[ti] OR ”deep learning”[ti]) NOT (”CT”[ti] OR ”compute*”[ti] OR ”tomogr”[ti] OR ”SPECT”[ti] OR ”MR”[ti] OR ”MRI”[ti] OR ”magnetic”[ti])

Web of Science

TS=(”Coronary Angiography” OR ”Coronary Angiographies” OR ”coronary angiogram*”) AND TI=(”Artificial Intelligence” OR ”AI” OR ”computer assisted Image Processing” OR ”automatic image analysis” OR ”machine learning” OR ”deep learning”) NOT TI=(”CT” OR ”compute*” OR ”tomogr” OR ”SPECT” OR ”MR” OR ”MRI” OR ”magnetic”)

IEEE

”All Metadata”:coronary angiography AND (”All Metadata”:artificial intelligence OR ”All Metadata”:deep learning OR ”All Metadata”:machine learning) NOT (”Document Title”:”CT” OR ”Document Title”:”Computed tomography*”)

EMBASE

(’artificial intelligence’:ti,ab,kw OR ’artificial intelligence’/exp OR ’deep learning’:ti,ab,kw OR ’deep learning’/exp OR ’machine learning’:ti,ab,kw OR ’machine learning’/exp) AND (’coronary angiography’/exp OR ’coronary angiography’:ti,ab,kw) NOT (’computer assisted tomography’/exp OR ’nuclear magnetic resonance imaging’/exp)

**Supplement B. Included studies**

1. Nasr-Esfahani E, Samavi S, Karimi N, Soroushmehr SMR, Ward K, Jafari MH, et al., editors. Vessel extraction in X-ray angiograms using deep learning. In: 2016 38th Annual International Conference of the IEEE Engineering in Medicine and Biology Society (EMBC); 2016 Aug 16–20; Orlando, FL. IEEE; 2016. p. 6433–6.
2. Jo K, Kweon J, Kim Y-H, Choi J. Segmentation of the main vessel of the left anterior descending artery using selective feature mapping in coronary angiography. IEEE Access. 2019;7:919–30.
3. Plourde M, Luc D, editors. Multi-scale classification approach for coronary artery detection from X-ray angiography. In: 2012 11th International Conference on Information Science, Signal Processing and their Applications (ISSPA); 2012 Jul 2–5; Montreal, QC. IEEE; 2012. p. 1197–200.
4. Zhao L, Li D, Chen J, Wan T, editors. Automated coronary tree segmentation for X-ray angiography sequences using fully-convolutional neural networks. In: 2018 IEEE Visual Communications and Image Processing (VCIP); 2018 Dec 9–12; Taichung. IEEE; 2018. p. 1–4.
5. Cruz-Aceves I, Cervantes-Sanchez F, Avila-Garcia MS. A novel multiscale Gaussian-matched filter using neural networks for the segmentation of X-ray coronary angiograms. J Healthc Eng. 2018;2018:5812059.
6. Fan J, Yang J, Wang Y, Yang S, Ai D, Huang Y, et al. Multichannel fully convolutional network for coronary artery segmentation in X-ray angiograms. IEEE Access. 2018;6:44635–43.
7. Nasr-Esfahani E, Karimi N, Jafari MH, Soroushmehr SMR, Samavi S, Nallamothu BK, et al. Segmentation of vessels in angiograms using convolutional neural networks. Biomed Signal Process Control. 2018;40:240–51.
8. Yang S, Yang J, Wang Y, Yang Q, Ai D, Wang Y. Automatic coronary artery segmentation in X-ray angiograms by multiple convolutional neural networks. In: Proceedings of the 3rd International Conference on Multimedia and Image Processing; 2018 Jan 1–3; Guiyang. ACM; 2018. p. 31–5.
9. Ma B, Liu S, Zhi Y, Song Q. Flow-based self-supervised pixel embedding for image segmentation. arXiv [Preprint]. 2019. Available from: https://arxiv.org/abs/1902.11137
10. Cervantes-Sanchez F, Cruz-Aceves I, Hernandez-Aguirre A, Hernandez-Gonzalez MA, Solorio-Meza SE. Automatic segmentation of coronary arteries in X-ray angiograms using multiscale analysis and artificial neural networks. Appl Sci. 2019;9(24):5447.
11. Yang S, Kweon J, Roh JH, Lee JH, Kang H, Park LJ, et al. Deep learning segmentation of major vessels in X-ray coronary angiography. Sci Rep. 2019;9(1):16897.
12. Yang S, Kweon J, Kim YH. Major vessel segmentation on X-ray coronary angiography using deep networks with a novel penalty loss function. In: Proceedings of the Korean Institute of Electrical Engineers Annual Conference; 2019.
13. Yu F, Zhao J, Gong Y, Wang Z, Li Y, Yang F, et al. Annotation-free cardiac vessel segmentation via knowledge transfer from retinal images. In: Lecture Notes in Computer Science. Cham: Springer; 2019. p. 714–22.
14. Hao D, Liu Y, Qin B. Learning saliently temporal-spatial features for X-ray coronary angiography sequence segmentation. In: Jiang X, Hwang J-N, editors. Proceedings of the 11th International Conference on Digital Image Processing (ICDIP); 2019 Aug 14–17; Guangzhou. SPIE; 2019.
15. Li R-Q, Bian G-B, Zhou X-H, Xie X, Ni Z-L, Hou Z, et al., editors. CAU-net: a novel convolutional neural network for coronary artery segmentation in digital subtraction angiography. In: Neural Information Processing. Cham: Springer; 2020. p. 311–22.
16. Jun TJ, Kweon J, Kim YH, Kim D. T-Net: nested encoder-decoder architecture for the main vessel segmentation in coronary angiography. Neural Netw. 2020;128:216–33.
17. Xian Z, Wang X, Yan S, Yang D, Chen J, Peng C, et al. Main coronary vessel segmentation using deep learning in smart medical. Math Probl Eng. 2020;2020:1–9.
18. Lourenço-Silva J, Menezes MN, Rodrigues T, Silva B, Pinto FJ, Oliveira AL, et al., editors. Encoder-decoder architectures for clinically relevant coronary artery segmentation. In: Computational Advances in Bio and Medical Sciences. Cham: Springer; 2022. p. 49–60.
19. Mulay S, Ram K, Murugesan B, Sivaprakasam M, editors. Style transfer-based coronary artery segmentation in X-ray angiogram. In: 2021 IEEE/CVF International Conference on Computer Vision Workshops (ICCVW); 2021 Oct 11–17; Montreal, QC. IEEE; 2021. p. 4296–300.
20. Hamdi R, Kerkeni A, Bedoui MH, Ben Abdallah A. CAS-net: a novel coronary artery segmentation neural network. In: ESANN 2021 Proceedings; Louvain-la-Neuve (Belgium): Ciaco-i6doc.com; 2021. p. 173–8.
21. Hao D, Ding S, Qiu L, Lv Y, Fei B, Zhu Y, et al. Sequential vessel segmentation via deep channel attention network. Neural Netw. 2020;128:172–87.
22. Iyer K, Najarian CP, Fattah AA, Arthurs CJ, Soroushmehr SMR, Subban V, et al. AngioNet: a convolutional neural network for vessel segmentation in X-ray angiography. Sci Rep. 2021;11(1):18066.
23. Jiang Z, Ou C, Qian Y, Rehan R, Yong A. Coronary vessel segmentation using multiresolution and multiscale deep learning. Inform Med Unlocked. 2021;24:100590.
24. Samuel PM, Veeramalai T. VSSC Net: vessel specific skip chain convolutional network for blood vessel segmentation. Comput Methods Programs Biomed. 2021;198:105769.
25. Wan T, Chen J, Zhang Z, Li D, Qin Z. Automatic vessel segmentation in X-ray angiogram using spatio-temporal fully-convolutional neural network. Biomed Signal Process Control. 2021;68:102731.
26. Zhou Y, Chang W, Song J, Guo H, Wang J, Chen Y. Semi-supervised deep learning of vessel segmentation in coronary angiography. In: 2021 IEEE 6th International Conference on Signal and Image Processing (ICSIP); 2021 Aug 27–29; Nanjing. IEEE; 2021. p. 194–8.
27. Zhu X, Cheng Z, Wang S, Chen X, Lu G. Coronary angiography image segmentation based on PSPNet. Comput Methods Programs Biomed. 2021;200:105897.
28. Tu S, Ding D, Chang Y, Li C, Wijns W, Xu B. Diagnostic accuracy of quantitative flow ratio for assessment of coronary stenosis significance from a single angiographic view: a novel method based on bifurcation fractal law. Catheter Cardiovasc Interv. 2021;97(Suppl 2):1040–7.
29. Busto L, González-Nóvoa JA, Juan-Salvadores P, Jiménez V, Íñiguez A, Veiga C, et al., editors. Using deep learning on X-ray orthogonal coronary angiograms for quantitative coronary analysis. In: Medical Image Understanding and Analysis. Cham: Springer; 2022. p. 287–97.
30. Wang L, Liang DX, Yin XL, Qiu J, Yang ZY, Xing JH, et al. Coronary artery segmentation in angiographic videos using a 3D–2D CE-Net. arXiv [Preprint]. 2020. Available from: <https://arxiv.org/abs/2001.03133>
31. Hamdi R, Kerkeni A, Bedoui MH, Ben Abdallah A, et al., editors. Res-GAN: residual generative adversarial network for coronary artery segmentation. In: Intelligent Data Engineering and Automated Learning – IDEAL 2022. Cham: Springer; 2022. p. 473–83
32. Gao Y, Zhang L, Zhao J, Jiang Z. Improved U-Net with channel and spatial attention for coronary angiography segmentation. In: 2022 16th ICME International Conference on Complex Medical Engineering (CME); 2022. p. 123–6.
33. He H, Banerjee A, Beetz M, Choudhury RP, Grau V. Semi-supervised coronary vessels segmentation from invasive coronary angiography with connectivity-preserving loss function. In: 2022 IEEE 19th International Symposium on Biomedical Imaging (ISBI); 2022. p. 1–5.
34. Song S, Xiao R, Lu T, Zhu H. Coronary artery segmentation from X-ray angiographic images using width-aware U-Net. In: 2022 2nd International Conference on Computer Graphics, Image and Virtualization (ICCGIV); 2022. p. 111–4.
35. Tao X, Dang H, Zhou X, Xu X, Xiong D. A lightweight network for accurate coronary artery segmentation using X-ray angiograms. Front Public Health. 2022;10:892418
36. Yin ZX, Xu HM. An unsupervised image segmentation algorithm for coronary angiography. BioData Min. 2022;15(1):27.
37. Nobre Menezes M, Silva B, Silva JL, Rodrigues T, Marques JS, Guerreiro C, et al. Segmentation of X-ray coronary angiography with an artificial intelligence deep learning model: impact in operator visual assessment of coronary stenosis severity. Catheter Cardiovasc Interv. 2023;102(4):631–40.
38. Menezes MN, Silva JL, Silva B, Rodrigues T, Guerreiro C, Guedes JP, et al. Coronary X-ray angiography segmentation using artificial intelligence: a multicentric validation study of a deep learning model. Int J Cardiovasc Imaging. 2023;39(7):1385–96.
39. Dang H, Zhang Y, Qi X, Zhou W, Sun M, et al., editors. Lightvessel: exploring lightweight coronary artery vessel segmentation via similarity knowledge distillation. In: ICASSP 2023 – 2023 IEEE International Conference on Acoustics, Speech and Signal Processing (ICASSP); 2023 Jun 4–10. IEEE; 2023. p. 1366–70.
40. Park J, Kweon J, Kim YI, Back I, Chae J, Roh JH, et al. Selective ensemble methods for deep learning segmentation of major vessels in invasive coronary angiography. Med Phys. 2023;50(12):7822–39.
41. Shi T, Ding X, Zhou W, Pan F, Yan Z, Bai X, et al. Affinity feature strengthening for accurate, complete and robust vessel segmentation. IEEE J Biomed Health Inform. 2023;27(8):4006–17.
42. Meng YH, Du ZL, Zhao C, Dong MH, Pienta D, Tang JS, et al. Automatic extraction of coronary arteries using deep learning in invasive coronary angiograms. Technol Health Care. 2023;31(6):2303–17.
43. Shen Y, Chen Z, Tong J, Jiang N, Ning Y. DBCU-Net: deep learning approach for segmentation of coronary angiography images. Int J Cardiovasc Imaging. 2023;39(8):1571–9.
44. Zhang H, Gao Z, Zhang D, Hau WK, Zhang H. Progressive perception learning for main coronary segmentation in X-ray angiography. IEEE Trans Med Imaging. 2023;42(3):864–79.
45. Bao F, Zhao Y, Zhang X, Zhang Y, Ning Y. SARC-UNet: a coronary artery segmentation method based on spatial attention and residual convolution. Comput Methods Programs Biomed. 2024;255:108353.
46. Yao TC, Lin KW, Lee CK, Tseng PH, Lee CR, et al., editors. Training sequential CAG segmentation models without labeled CAG video data. In: 2024 IEEE 48th Annual Computers, Software, and Applications Conference (COMPSAC); 2024 Jul 2–4. IEEE; 2024. p. 707–12.
47. Wang Z, Liu J, et al., editors. Semi-supervised learning and focal masking for vessel segmentation in X-ray coronary angiography. In: 2024 International Conference on Machine Learning and Applications (ICMLA); 2024 Dec 18–20. IEEE; 2024. p. 172–7.
48. Kim B, Oh Y, Wood BJ, Summers RM, Ye JC. C-DARL: contrastive diffusion adversarial representation learning for label-free blood vessel segmentation. Med Image Anal. 2024;91:102898.
49. Lee CK, Hong JW, Wu CL, Hou JM, Lin YA, Huang KC, et al. Real-time coronary artery segmentation in CAG images: a semi-supervised deep learning strategy. Artif Intell Med. 2024;153:102263.
50. Wahid F, Ma Y, Sheikh R, Aamir M, et al., editors. An enhanced convolutional neural network based on L1 regularization for segmentation of coronary arteries in X-ray angiograms. In: 2024 5th International Conference on Advancements in Computational Sciences (ICACS); 2024 Feb 19–20. IEEE; 2024. p. 1–6.
51. Velvizhi R, Ankayarkanni B, et al., editors. Segmenting stenotic regions from coronary angiograms using mask region-based convolutional neural network. In: 2024 5th International Conference on Data Intelligence and Cognitive Informatics (ICDICI); 2024 Nov 18–20. IEEE; 2024. p. 164–9.
52. Li S, Fan Y, et al., editors. Coronary artery segmentation in X-ray angiography based on deep learning approach. In: 2024 43rd Chinese Control Conference (CCC); 2024 Jul 28–31. IEEE; 2024. p. 3892–7.
53. Zeng Y, Liu H, Hu J, Zhao Z, She Q. Pretrained subtraction and segmentation model for coronary angiograms. Sci Rep. 2024;14(1):19888.
54. Ren H, Li D, Jing F, Zhang X, Tian X, Xie S, et al. LASF: a local adaptive segmentation framework for coronary angiogram segments. Health Inf Sci Syst. 2025;13(1):8.
55. Deng H, Fang T, Min X, et al., editors. Multi-scale across attention incorporated network for X-ray coronary vessel segmentation. In: ICASSP 2025 – 2025 IEEE International Conference on Acoustics, Speech and Signal Processing (ICASSP); 2025 Apr 6–11. IEEE; 2025. p. 1225–9.
56. He HR, Banerjee A, Choudhury RP, Grau V. Deep learning-based coronary vessels segmentation in X-ray angiography using temporal information. Med Image Anal. 2025;102:102860.
57. Yu J, Jiang Q. Asymmetric up-down sampling and complementary-fusion network for coronary artery segmentation on coronary angiography images. Biomed Signal Process Control. 2025;105:105934.
58. Gao Y, Ai D, Cao K, Song H, Fan J, Xiao D, et al. Spatio-temporal correspondence attention network for vessel segmentation in X-ray coronary angiography. Biomed Signal Process Control. 2025;99:104182.
59. Mahendiran T, Thanou D, Senouf O, Jamaa Y, Fournier S, De Bruyne B, et al. AngioPy Segmentation: an open-source, user-guided deep learning tool for coronary artery segmentation. Int J Cardiol. 2025;418:132598.
60. Nobre Menezes M, Lourenço-Silva J, Silva B, Rodrigues T, Francisco ARG, Carrilho Ferreira P, et al. Development of deep learning segmentation models for coronary X-ray angiography: quality assessment by a new global segmentation score and comparison with human performance. Rev Port Cardiol. 2022;41(12):1011–21.
61. Kumar S, Amutha. Automated classification of coronary artery disease using discrete wavelet transform and back propagation neural network. Sci Res Essays. 2014;9(10):440–51.
62. Au B, Shaham U, Dhruva S, Bouras G, Cristea E, Md AL, et al. Automated characterization of stenosis in invasive coronary angiography images with convolutional neural networks. arXiv [cs.CV]. 2018.
63. Du T, Liu X, Zhang H, Xu B. Real-time lesion detection of cardiac coronary artery using deep neural networks. In: 2018 International Conference on Network Infrastructure and Digital Content (IC-NIDC); 2018 Aug. IEEE; 2018. p. 157–61.
64. Zhang D, Yang G, Zhao S, Zhang Y, Zhang H, Li S, et al., editors. Direct quantification for coronary artery stenosis using multiview learning. In: Medical Image Computing and Computer Assisted Intervention – MICCAI 2019; 2019. Cham: Springer; 2019. p. 405–13.
65. Cong C, Kato Y, Vasconcellos HD, Lima J, Venkatesh B. Automated stenosis detection and classification in X-ray angiography using deep neural network. In: 2019 IEEE International Conference on Bioinformatics and Biomedicine (BIBM); 2019 Nov. IEEE; 2019. p. 2657–61.
66. Liu X, Du T, Zhang H, Sun C. Detection and classification of chronic total occlusion lesions using deep learning. In: 2019 41st Annual International Conference of the IEEE Engineering in Medicine and Biology Society (EMBC); 2019 Jul. IEEE; 2019. p. 865–8.
67. Chen S, Tang Y, Shi X, Zhang H, Xie L, Xu B. Convolution pyramid network: a classification network on coronary artery angiogram images. In: 2020 42nd Annual International Conference of the IEEE Engineering in Medicine & Biology Society (EMBC); 2020 Jul. IEEE; 2020. p. 1240–3.
68. Wu W, Zhang J, Xie H, Zhao Y, Zhang S, Gu L. Automatic detection of coronary artery stenosis by convolutional neural network with temporal constraint. Comput Biol Med. 2020;118:103657.
69. Danilov VV, Klyshnikov KY, Gerget OM, Kutikhin AG, Ganyukov VI, Frangi AF, et al. Real-time coronary artery stenosis detection based on modern neural networks. Sci Rep. 2021;11(1):7582.
70. Du T, Xie L, Zhang H, Liu X, Wang X, Chen D, et al. Training and validation of a deep learning architecture for the automatic analysis of coronary angiography. EuroIntervention. 2021;17(1):32–40.
71. Moon JH, Lee DY, Cha WC, Chung MJ, Lee KS, Cho BH, et al. Automatic stenosis recognition from coronary angiography using convolutional neural networks. Comput Methods Programs Biomed. 2021;198:105819.
72. Pang K, Ai D, Fang H, Fan J, Song H, Yang J. Stenosis-DetNet: sequence consistency-based stenosis detection for X-ray coronary angiography. Comput Med Imaging Graph. 2021;89:101900.
73. Yabushita H, Goto S, Nakamura S, Oka H, Nakayama M, Goto S. Development of novel artificial intelligence to detect the presence of clinically meaningful coronary atherosclerotic stenosis in major branch from coronary angiography video. J Atheroscler Thromb. 2021;28(8):835–43.
74. Freitas SA, Zeiser FA, Da Costa CA, De O Ramos G. DeepCADD: a deep learning architecture for automatic detection of coronary artery disease. In: 2022 International Joint Conference on Neural Networks (IJCNN); 2022. IEEE; 2022. p. 1–8.
75. Luque-Baena RM, Granados IR, Jimenez-Partinen A, Palomo EJ, Jimenez-Navarro M. Stenosis detection in coronary angiography images using deep learning models. In: 2022 IEEE International Conference on Metrology for Extended Reality, Artificial Intelligence and Neural Engineering (MetroXRAINE); 2022. IEEE; 2022. p. 22–7.
76. Ovalle-Magallanes E, Avina-Cervantes JG, Cruz-Aceves I, Ruiz-Pinales J. Hybrid classical–quantum convolutional neural network for stenosis detection in X-ray coronary angiography. Expert Syst Appl. 2022;189:116058.
77. Avram R, Olgin JE, Ahmed Z, Verreault-Julien L, Wan A, Barrios J, et al. CathAI: fully automated coronary angiography interpretation and stenosis estimation. NPJ Digit Med. 2023;6(1):9.
78. Haltiuk M, Czyjt M, Ciezobka W, Serwatka W, Galkowski J, Jarzab M, et al., editors. Automated stenosis detection in coronarography image data. In: 2023 IEEE EMBS Special Topic Conference on Data Science and Engineering in Healthcare, Medicine and Biology; 2023 Dec 7–9. IEEE; 2023. p. 109–12.
79. Wu H, Zhao J, Li J, Zeng Y, Wu W, Zhou Z, et al. One-stage detection without segmentation for multi-type coronary lesions in angiography images using deep learning. Diagnostics. 2023;13(18):2938.
80. Osama M, Kumar R, Shahid M, editors. Empowering cardiologists with deep learning YOLOv8 model for accurate coronary artery stenosis detection in angiography images. In: 2023 International Conference on IoT, Communication and Automation Technology (ICICAT); 2023 Jun 23–24. IEEE; 2023. p. 1–6.
81. Han T, Ai D, Li X, Fan J, Song H, Wang Y, et al. Coronary artery stenosis detection via proposal-shifted spatial-temporal transformer in X-ray angiography. Comput Biol Med. 2023;153:106546.
82. Moon IT, Kim SH, Chin JY, Park SH, Yoon CH, Youn TJ, et al. Accuracy of artificial intelligence-based automated quantitative coronary angiography compared to intravascular ultrasound: retrospective cohort study. JMIR Cardio. 2023;7:e44955.
83. Yue H, Yu W, Dong J, Lai Y, Wu Y, Zhao H, et al. Feasibility of the anchor-free deep learning method in coronary stenosis automatic detection. J Interv Cardiol. 2024;2024:1–9.
84. Kim D, Kim SH, Chu HW, Kang SH, Yoon CH, Youn TJ, et al. Validation of artificial intelligence-based quantitative coronary angiography. Digit Health. 2024;10:20552076241230254.
85. Labrecque Langlais É, Corbin D, Tastet O, Hayek A, Doolub G, Mrad S, et al. Evaluation of stenoses using AI video models applied to coronary angiography. NPJ Digit Med. 2024;7(1):138.
86. In Kim Y, Roh JH, Kweon J, Kwon H, Chae J, Park K, et al. Artificial intelligence-based quantitative coronary angiography of major vessels using deep learning. Int J Cardiol. 2024;405:131945.
87. Jiménez-Partinen A, Thurnhofer-Hemsi K, Rodríguez-Capitán J, Molina-Ramos AI, Palomo EJ. Coronary artery disease classification with different lesion degree ranges based on deep learning. IEEE Access. 2024;12:69229–39.
88. Wang T, Su X, Liang Y, Luo X, Hu X, Xia T, et al. Integrated deep learning model for automatic detection and classification of stenosis in coronary angiography. Comput Biol Chem. 2024;112:108184.
89. Begum F, Uddin YMR, editors. Enhancing cardiovascular diagnostics with YOLOv8: a deep learning approach to focal anomaly detection in angiogram imaging. In: 2024 4th International Conference on Ubiquitous Computing and Intelligent Information Systems (ICUIS); 2024 Dec 12–13. IEEE; 2024. p. 123–7.
90. Rostami A, Fouladi F, Sajedi H, editors. Segmentation of coronary artery stenosis in X-ray angiography using Mamba models. In: 2024 14th International Conference on Computer and Knowledge Engineering (ICCKE); 2024 Nov 19–20. IEEE; 2024. p. 95–100.
91. Duan H, Yi S, Ren Y. DCA-YOLOv8: a novel framework combined with AICI loss function for coronary artery stenosis detection. Sensors. 2024;24(24):9501.
92. Chae J, Kweon J, Park GM, Park S, Yoon HJ, Lee CH, et al. Enhancing quantitative coronary angiography (QCA) with advanced artificial intelligence: comparison with manual QCA and visual estimation. Int J Cardiovasc Imaging. 2025;41(3):559–68.
93. Eschen CK, Banasik K, Dahl AB, Chmura PJ, Bruun-Rasmussen P, Pedersen F, et al. Automated stenosis estimation of coronary angiographies using end-to-end learning. Int J Cardiovasc Imaging. 2025;41(3):441–52.
94. Kim Y, Yoon HJ, Suh J, Kang SH, Lim YH, Jang DH, et al. Artificial intelligence-based fully automated quantitative coronary angiography vs optical coherence tomography-guided PCI: the FLASH trial. JACC Cardiovasc Interv. 2025;18(2):187–97.
95. Kaba S, Haci H, Isin A, Ilhan A, Conkbayir C. The application of deep learning for the segmentation and classification of coronary arteries. Diagnostics. 2023;13(13):2192.
96. Zhou C, Dinh TV, Kong H, Yap J, Yeo KK, Lee HK, et al. Automated deep learning analysis of angiography video sequences for coronary artery disease. arXiv [eess.IV]. 2021. Available from: https://arxiv.org/abs/2111.03700
97. Zhao C, Vij A, Malhotra S, Tang J, Tang H, Pienta D, et al. Automatic extraction and stenosis evaluation of coronary arteries in invasive coronary angiograms. Comput Biol Med. 2021;136:104667.
98. Ling H, Chen B, Guan R, Xiao Y, Yan H, Chen Q, et al. Deep learning model for coronary angiography. J Cardiovasc Transl Res. 2023;16(4):896–904.
99. Zhang H, Zhang D, Gao Z, Zhang H, editors. Joint segmentation and quantification of main coronary vessels using dual-branch multi-scale attention network. In: Medical Image Computing and Computer Assisted Intervention – MICCAI 2021; 2021 Sept. Cham: Springer; 2021. p. 165–74.
100. Ben-Assa E, Abu Salman A, Cafri C, Roguin A, Hellou E, Koifman E, et al. Performance of a novel artificial intelligence software developed to derive coronary fractional flow reserve values from diagnostic angiograms. Coron Artery Dis. 2023;34(8):533–41.
101. Arefinia F, Aria M, Rabiei R, Hosseini A, Ghaemian A, Roshanpoor A. Non-invasive fractional flow reserve estimation using deep learning on intermediate left anterior descending coronary artery lesion angiography images. Sci Rep. 2024;14(1):1818.
102. Zhang D, Liu X, Wang A, Zhang H, Yang G, Zhang H, et al. Constraint-aware learning for fractional flow reserve pullback curve estimation from invasive coronary imaging. IEEE Trans Med Imaging. 2024;43(12):4091–104.
103. Mineo R, Salanitri FP, Bellitto G, Kavasidis I, Di Filippo O, Millesimo M, et al. A convolutional-transformer model for FFR and iFR assessment from coronary angiography. IEEE Trans Med Imaging. 2024;43(8):2866–77.
104. Oliveira C, Vilela M, Marques JS, Jorge C, Rodrigues T, Francisco AR, et al. Non-invasive derivation of instantaneous free-wave ratio from invasive coronary angiography using a new deep learning artificial intelligence model and comparison with human operators’ performance. Int J Cardiovasc Imaging. 2025. doi:10.1007/s10554-024-02947-1
105. Omori H, Kawase Y, Mizukami T, Tanigaki T, Hirata T, Okubo M, et al. Diagnostic performance of artificial intelligence-based angiography-derived non-hyperemic pressure ratio using pressure wire as reference. Circ J. 2025;89(3):323–30.
106. Menezes MN, Silva JL, Silva B, de Oliveira RM, Rodrigues T, Oliveira AL, et al. Coronary physiology instantaneous wave-free ratio (iFR) derived from X-ray angiography using artificial intelligence deep learning models: a pilot study. J Invasive Cardiol. 2024;36(3):E132–8.
107. De Filippo O, Mineo R, Millesimo M, Wanha W, Proietto Salanitri F, Greco A, et al. Non-invasive physiological assessment of intermediate coronary stenoses from plain angiography through artificial intelligence: the STARFLOW system. Eur Heart J Qual Care Clin Outcomes. 2024;9:345–53.
108. Roguin A, Abu Dogosh A, Feld Y, Konigstein M, Lerman A, Koifman E. Early feasibility of automated artificial intelligence angiography-based fractional flow reserve estimation. Am J Cardiol. 2021;139:8–14.
109. Bransby KM, Tufaro V, Çap M, Slabaugh G, Bourantas C, Zhang Q, editors. 3D coronary vessel reconstruction from bi-plane angiography using graph convolutional networks. In: 2023 IEEE 20th International Symposium on Biomedical Imaging (ISBI); 2023 Apr 18–21; Cartagena, Colombia. Piscataway: IEEE; 2023. p. 1–5.
110. Yan J, Fu Z, Fu Z, Guan Y, editors. A 3D/2D coronary artery registration method based on deep reinforcement learning. In: 2023 3rd International Conference on Computer, Control and Robotics (ICCCR); 2023 Mar 24–26; Shanghai, China. Piscataway: IEEE; 2023. p. 228–32.
111. Li C, Qiao Y, Yu W, Li Y, Chen Y, Fan Z, et al. AutoFOX: an automated cross-modal 3D fusion framework of coronary X-ray angiography and OCT. Med Image Anal. 2025;101:103432.
112. Kim HW, Noh SC, Kim SH, Chu HW, Jung CH, Kang SH. Effective descriptor extraction strategies for correspondence matching in coronary angiography images. Sci Rep. 2024;14(1):18630.
113. Kim C, Jeong D, Kim D, Ryu J, Cho K. A patient-specific registration of coronary angiogram-fluoroscopy by similarity-based transfer learning. IEEE Access. 2024;12:133509–20.
114. Fan J, Yang J, Wang Y, Yang S, Ai D, Huang Y, et al. Deep feature descriptor-based hierarchical dense matching for X-ray angiographic images. Comput Methods Programs Biomed. 2019;175:233–42.
115. Royer-Rivard R, Girard F, Dahdah N, Cheriet F. End-to-end deep learning model for cardiac cycle synchronization from multi-view angiographic sequences. In: 2020 42nd Annual International Conference of the IEEE Engineering in Medicine & Biology Society (EMBC); 2020 Jul 20–24; Montreal, QC, Canada. Piscataway: IEEE; 2020. p. 4442–5.
116. Dinescu AD, Miron R, Itu LM, Plajer IC, Turcea A, editors. XCAE: deep neural network for X-ray coronary angiograms quality enhancement. In: 2023 IEEE 28th International Conference on Emerging Technologies and Factory Automation (ETFA); 2023 Sept 12–15; Sinaia, Romania. Piscataway: IEEE; 2023. p. 1–4.
117. Fang H, Li H, Song S, Pang K, Ai D, Fan J, et al. Motion-flow-guided recurrent network for respiratory signal estimation of X-ray angiographic image sequences. Phys Med Biol. 2020;65(24):245020.
118. Moalla H, Ghrab A, Hamed BB, Bahloul A, Abid L, editors. Exploiting pre-trained architectures for dual-stream classification of LCA-RCA in a private AngioData. In: 2023 International Conference on Innovations in Intelligent Systems and Applications (INISTA); 2023 Sept 20–23; Madrid, Spain. Piscataway: IEEE; 2023. p. 1–6.
119. Zhao C, Esposito M, Xu Z, Zhou W. HAGMN-UQ: hyper association graph matching network with uncertainty quantification for coronary artery semantic labeling. Med Image Anal. 2025;99:103374.
120. Zhao C, Xu Z, Hung GU, Zhou W. EAGMN: coronary artery semantic labeling using edge attention graph matching network. Comput Biol Med. 2023;166:107548.
121. Cobo M, Pérez-Rojas F, Gutiérrez-Rodríguez C, Heredia I, Maragaño-Lizama P, Yung-Manriquez F, et al. Novel deep learning method for coronary artery tortuosity detection through coronary angiography. Sci Rep. 2023;13(1):837.
122. Hatfaludi CA, Bunescu D, Ciuşdel CF, Serban A, Böse K, Oppel M, et al., editors. Deep learning-based detection of collateral circulation in coronary angiographies. In: 2023 IEEE 36th International Symposium on Computer-Based Medical Systems (CBMS); 2023 Jun 22–24; L’Aquila, Italy. Piscataway: IEEE; 2023. p. 1–6.
123. Avram R, Barrios JP, Abreau S, Goh CY, Ahmed Z, Chung K, et al. Automated assessment of cardiac systolic function from coronary angiograms with video-based artificial intelligence algorithms. JAMA Cardiol. 2023;8(6):586–94.
124. Zhao Q, Li C, Chu M, Gutierrez-Chico JL, Tu S. Angiography-based coronary flow reserve: the feasibility of automatic computation by artificial intelligence. Cardiol J. 2023;30(3):369–78.
125. Liu L, Ding F, Shen Y, Tu S, Yang J, Zhao Q, et al. Automatic assessment of collaterals physiology in chronic total occlusions by means of artificial intelligence. Cardiol J. 2023;30(5):685–95.
126. Pérez-Martínez S, Fernández-Cisnal A, Pérez-Pelegrí M, García-Blas S, Miñana G, Valero E, et al., editors. Deep learning-based predictive model for revascularization of chronic total occlusions on angiographic imaging. In: 2023 45th Annual International Conference of the IEEE Engineering in Medicine & Biology Society (EMBC); 2023 Jul 24–27; Sydney, Australia. Piscataway: IEEE; 2023. p. 191–4.
127. Sun X, Belmpas T, Senouf O, Abbé E, Frossard P, De Bruyne B, et al., editors. Graph neural network-based future clinical events prediction from invasive coronary angiography. In: 2024 IEEE International Symposium on Biomedical Imaging (ISBI); 2024 May 27–30; Athens, Greece. Piscataway: IEEE; 2024. p. 1–4.
128. Dorina T, Ortal Yona S, Omar R, Emmanuel A, Pascal F, Farhang A, et al. Predicting future myocardial infarction from angiographies with deep learning. Adv Neural Inf Process Syst. 2021;34:1234–45.
129. Mahendiran T, Thanou D, Senouf O, Meier D, Dayer N, Aminfar F, et al. Deep learning-based prediction of future myocardial infarction using invasive coronary angiography: a feasibility study. Open Heart. 2023;10(1):e002288.
130. Sievering ID, Senouf O, Mahendiran T, Nanchen D, Fournier S, Muller O, et al. Anatomy-informed multimodal learning for myocardial infarction prediction. IEEE Open J Eng Med Biol. 2024;5:837–45.
131. Chang SS, Rostami B, LoRusso G, Liu CH, Alkhouli M. Optimizing input selection for cardiac model training and inference: an efficient 3D CNN-based approach to automate coronary angiogram video selection. medRxiv [Preprint]. 2024:20240512. DOI:10.1101/2024.05.12.123456.
132. Zhang R, Qin B, Zhao J, Zhu Y, Lv Y, Ding S. Locating X-ray coronary angiogram keyframes via long short-term spatiotemporal attention with image-to-patch contrastive learning. IEEE Trans Med Imaging. 2024;43(1):51–63.
133. Ciusdel C, Turcea A, Puiu A, Itu L, Calmac L, Weiss E, et al. Deep neural networks for ECG-free cardiac phase and end-diastolic frame detection on coronary angiographies. Comput Med Imaging Graph. 2020;84:101749.
134. Ma H, Ambrosini P, van Walsum T, editors. Fast prospective detection of contrast inflow in X-ray angiograms with convolutional neural network and recurrent neural network. In: Medical Image Computing and Computer Assisted Intervention – MICCAI 2017; 2017 Sept 10–14; Quebec City, QC, Canada. Cham: Springer; 2017. p. 253–6

Supplementary Tables

| Supplementary Table 1. Characteristics of included frame selection studies |
| --- |
| \| No. \| Frame \| Study type \| Input type \| TASK \| Architecture \| Ground truth \| Training dataset \| Test dataset \| ICA indication \| Dataset origin \| Availabilityy \| \| --- \| --- \| --- \| --- \| --- \| --- \| --- \| --- \| --- \| --- \| --- \| --- \| \| 1 \| Ma 2019 \| Model development \| Multi frame \| Contrast detection \| LSTM \| Manual \| 40 sequences \| 80 sequences \| - \| Clinical \| - \| \| 2 \| Ciusdel 2020 \| Model development \| Multi frame \| EDF detection \| CNN \| ECG \| 17.800 sequences \| 27900 sequences \| - \| clinical \| - \| \| 3 \| Chang 2024 \| Model development  external val. \| Multi frame \| High quality image identification \| CNN \| Manual \| 882 sequences \| 156 sequences (internal)  20388 sequences (external) \| - \| clinical \| - \| \| 4 \| Zhang 2024 \| Model development \| Multi frame \| Key-frame detection \| CNN-LSTM-transformer hybrid \| Manual \| 196 sequences \| 64 sequences \| - \| Clinical \| Model \| |
| CNN: convolutional neural network, EDF: end-diastolic frame, ICA: invasive coronary angiography. LSTM: long short-term memory network |
| Supplementary Table 2. Performance of frame selection models |
| \| No. \| FIRST AUTHOR \| Dataset \| Mean error (frames) \| accuracy \| Sensitivity \| Specificity \| NPV \| F1 \| AUC \| \| --- \| --- \| --- \| --- \| --- \| --- \| --- \| --- \| --- \| --- \| \| 1 \| Ma 2019 \| Internal \| 3.60 \| - \| - \| - \| - \| - \| - \| \| 2 \| Ciusdel 202 \| Internal \| - \| 0.982 \| 0.982 \| 0.981 \| 0.963 \| - \| - \| \| 3 \| Chang 2024 \| Internal \| - \| 0.960 \| 0.980 \| 0.950 \| - \| 0.920 \| 0.980 \| \|  \|  \| External \| - \| 0.920 \| 0.820 \| 0.93 \| - \| 0.710 \| 0.950 \| \| *4* \| Zhang 2024 \| Internal \| 5.16 \| - \| 0.842 \| - \| - \| 0.812 \| - \| |
| AUC: area under the receiver operating characteristic curve, NPV: Negative predictive value |

| Supplementary Table 3. Characteristics of included segmentation studies |
| --- |
| \| No \| First author \| Study type \| Input \| vessels \| Architecture \| Supervision \| Training dataset \| Test dataset \| ICA indication \| Dataset origin \| Availability \| \| --- \| --- \| --- \| --- \| --- \| --- \| --- \| --- \| --- \| --- \| --- \| --- \| \| 1 \| Plourde 2012 \| Model development \| Single frame \| WCT \| **S**VM \| Yes \| 20 images \| 50 images \| - \| Clinical \| - \| \| 2 \| Nasr-Esfahani 2016 \| Model development \| Single frame \| WCT \| CNN \| Yes \| 26 images \| 18 images \| - \| clinical \| - \| \| 3 \| Zhao 2018 \| Model development \| Multi frame \| WCT \| CNN \| Yes \| 50 sequences \| 10 sequences \| Suspected CAD \| Clinical \| - \| \| 4 \| Cruz-Aceves 2018 \| Model development \| Single frame \| WCT \| ANN \| Yes \| 50 images \| 50 images \| - \| Clinical \| - \| \| 5 \| Fan 2018 \| Model development \| Single frame \| WCT \| CNN \| Yes \| 130 images \| 18 images \| - \| clinical \| - \| \| 6 \| Jo 2018 \| Model development \| Single frame \| LAD \| CNN \| Yes \| 200 images \| 1787 images \| - \| clinical \| - \| \| 7 \| Nasr-Esfahani 2018 \| Model development \| Single frame \| WCT \| CNN \| Yes \| 33 images \| 11 images \| - \| clinical \| - \| \| 8 \| Yang 2018 \| Model development \| Single frame \| WCT \| CNN \| Yes \| 50 images \| 10 images \| - \| clinical \| - \| \| 9 \| Ma 2019 \| Model development \| Single frame \| WCT \| CNN \| Yes \| 582 images \| 145 images \| - \| Unknown \| - \| \| 10 \| Cervantes-Sanchez 2019 \| Model development \| Single frame \| WCT \| ANN \| Yes \| 100 images \| 30 images \| - \| Public \| Dataset \| \| 11 \| Yang-1 2019 \| Model development, external val. \| Single frame \| LAD, LCx, RCA \| CNN \| Yes \| 2621 images \| 1981 images (internal)  181 images (external) \| - \| clinical \| - \| \| 12 \| Yang-2 2019 \| Model development \| Single frame \| LAD, LCx, RCA \| CNN \| Yes \| 4458 images \| 3343 images \| - \| Clinical \| - \| \| 13 \| Yu 2019 \| Model development \| Single frame \| WCT \| GAN \| Semi \| 40 fundus images (annotated),  765 ICA images (unannotated) \| 328 ICA images \| - \| Clinical and public \| Dataset (partial) \| \| 14 \| Hao 2019 \| Model development \| Multi frame \| WCT \| CNN \| Yes \| 213 sequences \| 37 sequences \| - \| clinical \| - \| \| 15 \| Wang 2020 \| Model development \| Multi frame \| WCT \| CNN \| Yes \| 142 sequences \| 28 sequences \| - \| clinical \| - \| \| 16 \| Li 2020 \| Model development \| Single frame \| WCT \| CNN \| Yes \| 337 images \| 201 images \| - \| clinical \| - \| \| 17 \| jun-20 \| Model development \| Single frame \| LAD, LCx, RCA \| CNN \| Yes \| 3760 images \| 940 images \| - \| clinical \| - \| \| 18 \| Xian 2020 \| Model development \| Single frame \| LAD, LCx, RCA \| CNN \| Yes \| 2880 images \| 320 images \| - \| Clinical \| - \| \| 19 \| Lourenço 2021 \| Model development \| Single frame \| WCT \| CNN \| Yes \| 237 images \| 33 images \| - \| Clinical \| Model \| \| 20 \| Mulay 2021 \| Model development \| Single frame \| WCT \| CNN \| Yes \| 134 images \| 30 images \| - \| Public \| Dataset \| \| 21 \| Hamdi 2021 \| Model development \| Single frame \| WCT \| CNN \| Yes \| 120 images \| 30 images \| - \| Clinical \| - \| \| 22 \| Hao 2021 \| Model development \| Multi frame \| WCT \| CNN + attention \| Yes \| 249 sequences \| 83 sequences \| - \| Clinical \| Dataset, model \| \| 23 \| Iyer 2021 \| Model development \| Single frame \| WCT \| CNN \| Yes \| 224 images \| 56 images \| Diagnostic ICA \| Clinical \| Model \| \| 24 \| Jiang 2021 \| Model development \| Single frame \| WCT \| CNN \| Yes \| 104 images \| 30 images \| - \| Public \| Dataset \| \| 25 \| Samuel 2021 \| Model development \| Single frame \| WCT \| CNN \| Yes \| 120 images \| 40 images \| - \| Clinical \| - \| \| 26 \| Wan 2021 \| Model development \| Multi frame \| WCT \| CNN \| Yes \| - \| - \| Diagnostic ICA \| Clinical \| - \| \| 27 \| Zhou 2021 \| Model development \| Single frame \| WCT \| CNN + GAN \| Semi \| 60 images (annotated),  50 images (unannotated) \| 10 images \| - \| Clinical \| - \| \| 28 \| Zhu 2021 \| Model development \| Single frame \| WCT \| CNN \| Yes \| 73 images \| 36 images \| - \| Clinical \| - \| \| 29 \| Tu 2021 \| Model development \| Single frame \| WCT \| CNN \| Yes \| - \| - \|  \| - \| - \| \| 30 \| Busto 2022 \| Model development \| Single frame \| WCT \| CNN \| Yes \| 10 images \| 20 images \| Planned PCI \| Clinical \| - \| \| 31 \| Hamdi 2022 \| Model development \| Single frame \| WCT \| CNN + GAN \| Yes \| 120 images \| 30 images \| - \| Clinical \| - \| \| 32 \| Gao 2022 \| Model development \| Single frame \| WCT \| CNN + attention \| Yes \| 119 images \| 19 images \| - \| Clinical \| - \| \| 33 \| He 2022 \| Model development \| Single frame \| WCT \| CNN \| Semi \| 48 images (annotated), 60 images (unannotated) \| 10 images \| - \| Clinical \| - \| \| 34 \| Song 2022 \| Model development \| Single frame \| WCT \| CNN \| Yes \| 232 images \| 57 images \| - \| - \| - \| \| 35 \| Tao 2022 \| Model development, external val. \| Single frame \| WCT \| CNN \| Yes \| 234 images \| 50 images (internal)  150 images (External) \| - \| Public and clinical \| Dataset partial \| \| 36 \| Yin 2022 \| Model development \| Single frame \| WCT \| CNN \| No \| - \| - \| - \| Clinical \| - \| \| 37 \| Nobre- Menezes 2022 \| Model development \| Single frame \| WCT \| CNN \| Yes \| 372 images \| 31 images \| Diagnostic ICA including FFR \| Clinical \| - \| \| 38 \| Nobre-Menezes-1 2023 \| Clinical evaluation \| Single frame \| WCT \| CNN \| Yes \| No training \| 117 images \| Diagnostic ICA including FFR \| Clinical \| - \| \| 39 \| Nobre-Menezes-2 2023 \| External validation \| Single frame \| WCT \| CNN \| Yes \| No training \| 117 images \| Diagnostic ICA including FFR \| clinical \| - \| \| 40 \| Dang 2023 \| Model development \| Single frame \| WCT \| CNN \| Yes \| 200 images \| 40 images \| - \| Clinical \| - \| \| 41 \| Park 2023 \| Model development, external val. \| Single frame \| WCT \| CNN \| Yes \| 5941 images \| 1485 images (internal)  556 mages (external) \| - \| Clinical \| - \| \| 42 \| Shi 2023 \| Model development, external val. \| Single frame \| WCT \| CNN \| Yes \| 84 images \| 42 images (internal)  20 images (external) \| - \| Clinical and public \| Dataset partial \| \| 43 \| Meng 2023 \| Model development \| Single frame \| WCT \| CNN \| Yes \| 486 images \| 130 images \| - \| Clinical \| - \| \| 44 \| Shen 2023 \| Model development \| Single frame \| WCT \| CNN \| Yes \| 30 images \| 20 images \| - \| Clinical \| - \| \| 45 \| Zhang 2023 \| Model development \| Single frame \| LAD, LCx, RCA \| CNN \| Yes \| 1124 images \| 125 images \| - \| clinical and public \| Dataset partial \| \| 46 \| Bao 2024 \| Model development \| Single frame \| WCT \| CNN \| Yes \| 201 images \| 22 images \| - \| Clinical \| - \| \| 47 \| Yao 2024 \| Model development \| Multi frame \| WCT \| Transformer \| Semi \| 58 sequences (unannotated),  50 images (annotated) \| 3 sequences \| - \| Public \| Dataset \| \| 48 \| Wang 2024 \| Model development \| Single frame \| WCT \| Transformer \| Semi \| 1200 images (major vessel annotated only) \| 300 images \| - \| Public \| Dataset \| \| 49 \| Kim 2024 \| Model development \| Single frame \| WCT \| GAN \| No \| 1533 images \| 278 images \| - \| Public \| Dataset \| \| 50 \| Lee 2024 \| Model development \| Single frame \| WCT \| CNN \| Semi \| 400 images (annotated), 8952 images (unannotated) \| 100 images \| - \| Clinical \| - \| \| 51 \| Wahid 2024 \| Model development \| Single frame \| WCT \| CNN \| Yes \| 100 images \| 30 images \| - \| Public \| Dataset \| \| 52 \| Velvizhi 2024 \| Model development \| Single frame \| WCT \| CNN \| Yes \| 1350 images \| 150 images \| - \| Public \| Dataset \| \| 53 \| Li 2024 \| Model development \| Single frame \| LAD, LCx, RCA \| CNN \| Yes \| 1000 images \| 200 images \| - \| Public \| Dataset \| \| 54 \| Zeng 2024 \| Model development \| Single frame \| WCT \| CNN \| Semi \| 56328 images (unannotated),  40 images (annotated) \| 10 images \| - \| Public and clinical \| Datasets and model \| \| 55 \| Ren 2025 \| Model development \| Single frame \| WCT \| CNN \| Yes \| 3000 images \| - \| - \| Public \| Dataset \| \| 56 \| Deng 2025 \| Model development \| Single frame \| WCT \| CNN + attention \| Yes \| 292 images \| - \| - \| clinical \| - \| \| 57 \| He 2025 \| Model development, external val. \| Multi frame \| WCT \| CNN \| Yes \| 255 sequences \| 68 sequences (internal)  60 sequences (external) \| - \| Public and clinical \| Dataset partial \| \| 58 \| Yu 2025 \| Model development, external val. \| Single frame \| WCT \| CNN \| Yes \| 600 images \| - (internal)  100 images (external) \| - \| Clinical \| - \| \| 59 \| Gao 2025 \| Model development \| Multi frame \| WCT \| CNN + attention \| Yes \| 24 sequences \| 6 sequences \| - \| clinical \| - \| \| 60 \| Mahendiran 2025 \| Model development, external val. \| Single frame \| WCT \| CNN \| Yes \| 2455 images \| 500 images (internal)  83 images (external) \| Stable CAD \| Clinical trial (FAME2) \| Dataset and model \| |
| ANN: artificial neural network, CAD: coronary artery disease, CNN: convolutional neural network, FFR: fractional flow reserve, GAN: generative adversarial network, ICA: invasive coronary angiography, LAD: left anterior descending artery, LCx: left circumflex artery, PCI: percutaneous coronary intervention, RCA: right coronary artery, SVM: support vector machine, WCT: whole coronary tree. |

| Supplementary Table 4. Performance metrics for segmentation models. |
| --- |
| \| No. \| FIRST AUTHOR \| Dataset \| Accuracy \| Precision \| sensitivity \| Specificity \| NPV \| DSC \| IOU \| AUC \| \| --- \| --- \| --- \| --- \| --- \| --- \| --- \| --- \| --- \| --- \| --- \| \| 1 \| Plourde 2012 \| Internal \| 0.9778 \| - \| - \| - \| - \| - \| - \| - \| \| 2 \| Nasr-Esfahani 2016 \| Internal \| 0.935 \| 0.967 \| 0.900 \| 0.970 \| 0.906 \| - \| - \| - \| \| 3 \| Zhao 2018 \| Internal \| 0.957 \| - \| 0.826 \| 0.960 \| - \| 0.880 \| - \| - \| \| 4 \| Cruz-Aceves 2018 \| Internal \| 0.957 \| - \| - \| - \| - \| - \| - \| 0.936 \| \| 5 \| Fan 2018 \| Internal \| 0.988 \| 0.868 \| 0.877 \| 0.995 \| - \| 0.873 \| - \| - \| \| 6 \| Jo 2018 \| Internal \| 0.984 \| - \| - \| - \| - \| 0.659 \| - \| - \| \| 7 \| Nasr-Esfahani 2018 \| Internal \| 0.979 \| - \| 0.868 \| 0.986 \| - \| 0.815 \| - \| - \| \| 8 \| Yang 2018 \| Internal \| - \| 0.830 \| 0.774 \| 0.993 \| - \| 0.801 \| - \| - \| \| 9 \| Ma 2019 \| Internal \| - \| - \| - \| - \| - \| 0.820 \| - \| - \| \| 10 \| Cervantes-Sanchez 2019 \| Internal \| 0.970 \| 0.740 \| 0.630 \| 0.988 \| - \| 0.686 \| - \| 0.978 \| \| 11 \| Yang-1 2019 \| Internal \| - \| 0.918 \| 0.921 \| - \| - \| 0.917 \| - \| - \| \|  \|  \| External \| - \| 0.904 \| 0.898 \| - \| - \| 0.896 \| - \| - \| \| 12 \| Yang-2 2019 \| Internal \| - \| 0.904 \| 0.898 \| - \| - \| 0.896 \| - \| - \| \| 13 \| Yu 2019 \| Internal \| - \| 0.913 \| 0.926 \| - \| - \| 0.919 \| - \| - \| \| 14 \| Hao 2019 \| Internal \| 0.953 \| 0.820 \| 0.829 \| - \| - \| 0.824 \| - \| - \| \| 15 \| Wang 2020 \| Internal \| - \| 0.860 \| 0.832 \| - \| - \| 0.844 \| - \| - \| \| 16 \| Li 2020 \| Internal \| - \| - \| 0.779 \| 0.994 \| - \|  \| 0.818 \| - \| \| 17 \| Jun 2020 \| Internal \| 0.992 \| - \| 0.886 \| 0.997 \| - \| 0.904 \| - \| - \| \| 18 \| Xian 2020 \| Internal \| - \| 0.896 \| 0.892 \| - \| - \| 0.900 \| - \| - \| \| 19 \| Lourenço 2021 \| Internal \| - \| 0.919 \| 0.92 \| - \| - \| 0.921 \| - \| - \| \| 20 \| Mulay 2021 \| Internal \| - \| - \| - \| - \| - \| 0.890 \| - \| - \| \| 21 \| Hamdi 2021 \| Internal \| 0.966 \| - \| 0.787 \| 0.976 \| - \| 0.717 \| - \| - \| \| 22 \| Hao 2021 \| Internal \| 0.969 \| 0.872 \| 0.790 \| 0.988 \| - \| 0.829 \| - \| - \| \| 23 \| Iyer 2021 \| Internal \| - \| 0.850 \| - \| - \| - \| 0.843 \| - \| - \| \| 24 \| Jiang 2021 \| Internal \| 0.973 \| 0.790 \| 0.789 \| 0.987 \| - \| 0.775 \| - \| - \| \| 25 \| Samuel 2021 \| Internal \| 0.970 \| - \| 0.773 \| 0.981 \| - \| - \| - \| 0.983 \| \| 26 \| Wan 2021 \| Internal \| 0.920 \| - \| 0.890 \| 0.920 \| - \| 0.900 \| - \| - \| \| 27 \| Zhou 2021 \| Internal \| 0.887 \| 0.891 \| 0.863 \| - \| - \| 0.876 \| - \| - \| \| 28 \| Zhu 2021 \| Internal \| 0.957 \| - \| 0.947 \| 0.951 \| - \|  \| - \| - \| \| 29 \| Tu 2021 \| Internal \| - \| - \| - \| - \| - \|  \| - \| - \| \| 30 \| Busto 2022 \| Internal \| 0.990 \| 0.890 \| 0.930 \| 0.990 \| - \| 0.940 \| 0.890 \| 0.970 \| \| 31 \| Hamdi 2022 \| Internal \| 0.966 \| 0.813 \| 0.811 \| 0.981 \| - \| 0.812 \| - \| - \| \| 32 \| Gao 2022 \| Internal \| 0.977 \| - \| 0.837 \| 0.9870 \| - \| 0.807 \| - \| 0.991 \| \| 33 \| He 2022 \| Internal \| - \| - \| 0.839 \| - \| - \| 0.817 \| - \| - \| \| 34 \| Song 2022 \| Internal \| 0.983 \| 0.714 \| - \| - \| - \| 0.773 \| - \| - \| \| 35 \| Tao 2022 \| Internal \| 0.973 \| - \| 0.877 \| 0.9790 \| - \|  \| - \| 0.991 \| \|  \|  \| External \| 0.970 \| - \| 0.898 \| 0.971 \| - \|  \| - \| 0.984 \| \| 36 \| Yin 2022 \| Internal \| 0.950 \| 0.900 \| 0.770 \| - \| - \| 0.830 \| - \| - \| \| 37 \| Nobre- Menezes 2022 \| Internal \| - \| - \| - \| - \| - \| 0.890 \| - \| - \| \| 38 \| Nobre-Menezes-1 2023 \| Clinical evaluation \| - \| - \| - \| - \| - \|  \| - \| - \| \| 39 \| Nobre-Menezes-2 2023 \| External \| 0.990 \| 0.949 \| 0.951 \| 0.990 \| 0.990 \| 0.948 \| - \| - \| \| 40 \| Dang 2023 \| Internal \| 0.9834 \| - \| 0.982 \| - \| - \| 0.779 \| 0.811 \| 0.994 \| \| 41 \| Park 2023 \| Internal \| - \| - \| - \| - \| - \| 0.930 \| - \| - \| \|  \|  \| External \| - \| - \| - \| - \| - \| 0.900 \| - \| - \| \| 42 \| Shi 2023 \| Internal \| - \| 0.810 \| 0.84 \| - \| - \| 0.820 \| - \| - \| \|  \| Shi 2023 \| External \| - \| 0.701 \| 0.787 \| - \| - \| 0.737 \| - \| - \| \| 43 \| Meng 2023 \| Internal \| - \| - \| 0.874 \| 0.995 \| - \| 0.894 \| - \| - \| \| 44 \| Shen 2023 \| Internal \| 0.985 \| 0.913 \| 0.847 \| - \| - \| 0.879 \| - \| 0.929 \| \| 45 \| Zhang 2023 \| Internal \| - \| - \| - \| - \| - \| 0.959 \| 0.926 \| - \| \| 46 \| Bao 2024 \| Internal \| 0.980 \| - \| - \| 0.993 \| - \| 0.772 \| - \| 0.984 \| \| 47 \| Yao 2024 \| Internal \| - \| 0.884 \| 0.845 \| - \| - \| 0.850 \| - \| - \| \| 48 \| Wang 2024 \| Internal \| - \| - \| - \| - \| - \| 0.793 \| - \| - \| \| 49 \| Kim 2024 \| Internal \| - \| 0.750 \| 0.602 \| - \| - \| 0.661 \| - \| - \| \| 50 \| Lee 2024 \| Internal \| - \| 0.884 \| 0.887 \| - \| - \| 0.884 \| - \| - \| \| 51 \| Wahid 2024 \| Internal \| 0.974 \| 0.753 \| 0.983 \| 0.998 \| - \| 0.710 \| - \| - \| \| 52 \| Velvizhi 2024 \| Internal \| - \| - \| 0.929 \| 0.966 \| - \| 0.978 \| - \| - \| \| 53 \| Li 2024 \| Internal \| 0.988 \| - \| 0.819 \| - \| - \| 0.822 \| - \| - \| \| 54 \| Zeng 2024 \| Internal \| 0.982 \| - \| 0.820 \| 0.993 \| - \| 0.828 \| - \| - \| \| 55 \| Ren 2025 \| Internal \| 0.960 \| 0.975 \| 0.940 \| - \| - \| 0.980 \| - \| - \| \| 56 \| Deng 2025 \| Internal \| 0.986 \| - \| - \| 0.993 \| - \| 0.893 \| - \| - \| \| 57 \| He 2025 \| Internal \| - \| 0.825 \| 0.843 \| - \| - \| 0.834 \| - \| - \| \|  \|  \| External \| - \| - \| 0.824 \| - \| - \| 0.785 \| - \| - \| \| 58 \| Yu 2025 \| Internal \| - \| - \| 0.682 \| 0.996 \| - \| 0.795 \| - \| - \| \|  \|  \| External \| - \| - \| - \| - \| - \| - \| - \| - \| \| 59 \| Gao 2025 \| Internal \| - \| 0.907 \| 0.878 \| - \| - \| 0.892 \| - \| - \| \| 60 \| Mahendiran 2025 \| Internal \| 0.998 \| 0.925 \| 0.999 \| - \| - \| 0.927 \| - \| - \| \|  \|  \| External \| 0.997 \| 0.922 \| 0.928 \| 0.999 \| - \| 0.924 \| - \| - \| |
| AUC: area under the ROC curve, DSC: Dice similarity coefficient, IOU: intersection over union, NPV: negative predictive value. |

| Supplementary Table 5. Characteristics of included lesion assessment studies |
| --- |
| \| No. \| First author \| Study type \| Input type \| Algoritme type \| Vessels \| Architecture \| Ground truth \| Training dataset \| Test dataset \| ICA indication \| Dataset origin \| Availability \| \| --- \| --- \| --- \| --- \| --- \| --- \| --- \| --- \| --- \| --- \| --- \| --- \| --- \| \| 1 \| Kumar 2014 \| Model development \| Single frame \| Segmentation based \| WCT \| ANN \| - \| 50 images \| - \| - \| Clinical \| - \| \| 2 \| Au 2018 \| Model development \| Single frame \| Segmentation based \| RCA only \| CNN \| QCA \| 784 images \| 138 images \| Stable CAD, UAP, NSTEMI \| Trial data (unspecified) \| - \| \| 3 \| Du 2018 \| Model development \| Single frame \| Bounding box \| WCT \| CNN \| PVA \| 2624 images \| 301 images \| - \| Clinical \| - \| \| 4 \| Zhang 2019 \| Model development \| Multi frame \| QCA prediction \| LAD, LCx, RCA \| CNN \| QCA \| 98 patients \| 12 patients \| - \| Clinical \| - \| \| 5 \| Cong 2019 \| Model development \| Multi frame \| Bounding box, classification \| WCT \| CNN \| QCA \| 131 patients \| 44 patients \| Stable CAD \| Trial data (CORE320) \| - \| \| 6 \| Liu 2019 \| Model development \| Single frame \| Bounding box, classification \| WCT \| CNN \| PVA \| 1909 images \| 150 images \| - \| Clinical \| - \| \| 7 \| Chen 2020 \| Model development \| Single frame \| Image classification \| WCT \| CNN \| PVA \| - \| - \| - \| Clinical \| - \| \| 8 \| Wu 2020 \| Model development \| Multi frame \| Bounding box \| WCT \| CNN \| PVA \| 123 sequences \| 24 sequences \| - \| Clinical \| - \| \| 9 \| Danilov 2021 \| Model development \| Single frame \| Bounding box \| WCT \| CNN \| PVA \| 7493 images \| 832 images \| Stable CAD \| clinical \| - \| \| 10 \| Du 2021 \| Model development \| Single frame \| Bounding box, Segmentation \| WCT \| CNN \| PVA \| 6239 images \| 1000 images \| - \| Clinical \| - \| \| 11 \| Moon 2021 \| Model development, external val. \| Multi frame \| Image classification \| WCT \| CNN, attention \| QCA \| 362 sequences \| 90 sequences (internal)  76 sequences \| - \| Clinical \| - \| \| 12 \| Pang 2021 \| Model development \| Multi frame \| Bounding box \| WCT \| CNN \| PVA \| 140 sequences \| 26 sequences \| - \| Clinical \| - \| \| 13 \| Yabushita 2021 \| Model development \| Multi frame \| Image classification \| WCT \| CNN \| PVA \| 1359 sequences \| 479 sequences \| Stable CAD and ACS \| clinical \| Model \| \| 14 \| Zhao 2021 \| Model development \| Single frame \| Segmentation based \| WCT \| CNN \| Manual segmentation \| 224 images \| 90 images \|  \| Clinical \| - \| \| 15 \| Zhou 2021 \| Model development \| Multi frame \| QCA prediction \| RCA \| CNN \| PVA \| 6985 images \| 1746 images \| - \| Clinical \|  \| \| 16 \| Zhang 2021 \| Model development \| Single frame \| QCA prediction \| LAD, LCx, RCA \| CNN, attention \| QCA \| 476 images \| 53 images \| - \| clinical \| - \| \| 17 \| Freitas 2022 \| Model development \| Single frame \| Bounding box, segmentation, image classification \| RCA only \| CNN \| PVA \| 2000 synthetic coronary segments, 118 images \| 12 images \| - \| Synthetic, clinical \| Dataset partial \| \| 18 \| Luque-Baena 2022 \| Model development \| Patch \| Per patch classification \| WCT \| CNN \| PVA \| 1135 patches \| 284 patches \| - \| Public \| Dataset \| \| 19 \| Ovalle-Magallanes 2022 \| Model development \| Patch \| Per patch classification \| WCT \| Hybrid classical-quantum CNN \| - \| 125 patches \| 125 patches \| - \| Public \| Dataset \| \| 20 \| Avram 2023 \| Model development, externa val. \| Multi frame \| Bounding box, stenosis percentage prediction \| LAD, LCx, RCA \| CNN \| PVA \| 84011 images \| 21.003 image (internal)  464 sequences (external) \| - \| Clinical \| - \| \| 21 \| Haltiuk 2023 \| Model development \| Single frame \| Bounding box \| WCT \| CNN \| - \| 97 images \| - \| - \| - \| - \| \| 22 \| Wu 2023 \| Model development \| Single frame \| Bounding box, classification \| WCT \| CNN \| PVA \| 3569 images \| 1649 images \| Stable CAD \| Clinical \| - \| \| 23 \| Osama 2023 \| Model development \| Single frame \| Bounding box \| WCT \| CNN \| - \| 198 images \| 38 images \|  \| Public \| Dataset \| \| 24 \| Han 2023 \| Model development \| Multi frame \| Bounding box \| WCT \| CNN-transformer hybrid \| PVA \| 175 sequences \| 58 sequences \| - \| clinical \| - \| \| 25 \| Moon 2023 \| Retrospective validation \| Single frame \| QCA prediction \| LAD, LCx, RCA \| CNN \| IVUS \| No training \| 50 patients \| IVUS guided PCI \| Clinical \| - \| \| 26 \| Kaba 2023 \| Model development \| Single frame \| Image classification \| LAD, LCx, RCA \| CNN \| QCA \| 136 images \| 34 images \| - \| clinical \| - \| \| 27 \| Ling 2023 \| Model development \| Single frame \| Bounding box, segmentation \| LAD, LCx, RCA \| CNN \| PVA \| 2980 images \| - \| - \| Clinical \| Model deployed online \| \| 28 \| Yue 2024 \| Model development \| Single frame \| Bounding box \| WCT \| CNN \| PVA \| 2507 images \| 279 images \| Elective PCI \| Clinical \| - \| \| 29 \| Kim 2024 \| External \| Single frame \| QCA prediction \| LAD, LCx, RCA \| CNN \| QCA \| No training \| 1017 images \| ACS and stable CAD \| Clinical \| - \| \| 30 \| Labreque Langlais 2023 \| Model development, externa val. \| Multi frame \| Bounding box, stenosis percentage prediction \| LAD, LCx, RCA \| Transformer \| PVA \| 37.518 sequences \| 6621 sequences (internal)  1267 sequences (external) \| - \| Clinical \| Model partial \| \| 31 \| In Kim 2024 \| Model development \| Single frame \| QCA prediction \| LM, LAD, LCx, RCA, PDA and PL \| CNN \| QCA \| 7658 images \| 676 images \| - \| Clinical \| - \| \| 32 \| Jimenez-Partinen 2024 \| Model development \| Patch \| Per patch classification \| WCT \| CNN \| PVA \| 4674 images \| 1169 images \| CCS and ACS \| clinical \| Dataset \| \| 33 \| Wang 2024 \| Model development \| Single frame \| Bounding box, classification \| RCA \| CNN \| PVA \| 1365 images \| 241 images \| - \| Clinical \| Model deployed online \| \| 34 \| Begum 2024 \| Model development \| Single frame \| Bounding box, classification \| WCT \| CNN \| - \| - \| - \| - \| Public \| Dataset \| \| 35 \| Rostami 2024 \| Model development \| Single frame \| Segmentation based \| WCT \| State-space based \| PVA \| 1200 images \| 300 images \| - \| Public \| Dataset \| \| 36 \| Duan 2024 \| Model development \| Single frame \| Bounding box \| WCT \| CNN, attention \| PVA \| 9012 images \| 832 images \| - \| Public \| Datasets and model \| \| 37 \| Chae 2025 \| External val. \| Single frame \| QCA prediction \| LAD, LCx, RCA \| CNN \| QCA \| No training \| 1076 images \| - \| Clinical \| - \| \| 38 \| Eschen 2025 \| Model development, external val. \| Multi frame \| Stenosis percentage prediction \| WCT \| CNN \| PVA \| 93.326 sequences \| 36497 sequences (internal)  4374 sequences (external) \| - \| Clinical \| - \| \| 39 \| Kim 2025 \| RCT \| Single frame \| QCA prediction \| LAD, LCx, RCA \| CNN \| OCT \| No training \| 200 patients \| Acute and elective PCI \| clinical \| - \| |
| Various papers describe ensembles of multiple models, all data mentioned is regarding the stenosis classification step of these algorithms  ACS: acute coronary syndrome, ANN: artificial neural network, CAD: coronary artery disease, CCS: chronic coronary syndrome, CNN: convolutional neural network, FFR: fractional flow reserve, GAN: generative adversarial network, ICA: invasive coronary angiography, LAD: left anterior descending artery, LCx: left circumflex artery, NSTEMI: non-ST elevation myocardial infarction, OCT: optical coherence tomography, PCI: percutaneous coronary intervention, PDA: posterior descending artery, PL: posterolateral branch, PVA: physician visual assessment, QCA: quantitative coronary angiography, RCT: randomized controlled trial, RCA: right coronary artery, SVM: support vector machine, UAP: unstable angina pectoris, WCT: whole coronary tree. |

| Supplementary Table 6. Performance metrics for lesion assessment models |
| --- |
| \| No. \| FIRST AUTHOR \| Dataset \| Accuracy \| Sensitivity \| Specificity \| precision \| NPV \| F1 \| MAE (mm) \| MAE (%) \| PCC \| AUC \| \| --- \| --- \| --- \| --- \| --- \| --- \| --- \| --- \| --- \| --- \| --- \| --- \| --- \| \| 1 \| Kumar 2014 \| Internal \| 0.885 \| - \| - \| - \| - \| - \| - \| - \| - \| - \| \| 2 \| Au 2018 \| Internal \| - \| - \| - \| - \| - \| - \| - \| - \| - \| 0.703 \| \| 3 \| Du 2018 \| Internal \| - \| 0.880 \| - \| 0.460 \| - \| - \| - \| - \| - \| - \| \| 4 \| Zhang 2019 \| Internal \| - \| - \| - \| - \| - \| - \| 1.27 \| - \| 0.891 \| - \| \| 5 \| Cong 2019 \| Internal \| 0.850 \| - \| - \| - \| - \| 0.770 \| - \| - \| - \| - \| \| 6 \| Liu 2019 \| Internal \| - \| 0.893 \| - \| 0.876 \| - \| 0.885 \| - \| - \| - \| - \| \| 7 \| Chen 2020 \| Internal \| - \| 0.944 \| - \| 0.949 \| - \| 0.946 \| - \| - \| - \| - \| \| 8 \| Wu 2020 \| Internal \| - \| 0.872 \| - \| 0.795 \| - \| 0.832 \| - \| - \| - \| - \| \| 9 \| Danilov 2021 \| Internal \| - \| - \| - \| 0.940 \| - \| 0.960 \| - \| - \| - \| - \| \| 10 \| Du 2021 \| Internal \| - \| - \| - \| - \| - \| 0.824 \|  \| - \| - \| - \| \| 11 \| Moon 2021 \| Internal \| 0.934 \| - \| - \| - \| - \| - \| - \| - \| - \| 0.971 \| \|  \|  \| External \| 0.917 \| - \| - \| - \| - \| - \| - \| - \| - \| 0.956 \| \| 12 \| Pang 2021 \| Internal \| - \| 0.822 \| - \| 0.949 \| - \| 0.881 \| - \| - \| - \| - \| \| 13 \| Yabushita 2021 \| Internal \| 0.600 \| 0.610 \| 0.600 \| - \| - \| 0.600 \| - \| - \| - \| - \| \| 14 \| Zhao 2021 \| Internal \| - \| 0.684 \| - \| 0.700 \| - \| - \| - \| - \| - \| - \| \| 15 \| Zhou 2021 \| Internal \| 0.803 \| - \| 0.729 \| - \| - \| - \| - \| 15.9 \| - \| - \| \| 16 \| Zhang 2021 \| Internal \| - \| - \| - \| - \| - \| - \| 1.30 \| - \| 0.913 \| - \| \| 17 \| Freitas 2022 \| Internal \| - \| 0.891 \| - \| 0.830 \| - \| - \| - \| - \| - \| - \| \| 18 \| Luque-Baena 2022 \| Internal \| 0.940 \| 0.900 \| 0.980 \| - \| - \| - \| - \| - \| - \| - \| \| 19 \| Ovalle-Magallanes 2022 \| Internal \| 0.918 \| 0.949 \| - \| 0.889 \| - \| - \| - \| - \| - \| - \| \| 20 \| Avram 2023 \| Internal \| - \| 0.745 \| 0.781 \| 0.461 \| 0.924 \| - \| - \| - \| - \| 0.862 \| \|  \|  \| External \| - \| 0.864 \| 0.673 \| - \| - \| - \| - \| - \| - \| 0.869 \| \|  \|  \| QCA \| - \| - \| - \| - \| - \| - \| - \| - \| - \| 0.775 \| \| 21 \| Haltiuk 2023 \| Internal \| - \| 0.511 \| - \| - \| - \| 0.450 \|  \|  \| 0.4792 \| 0.450 \| \| 22 \| Wu 2023 \| Internal \| - \| 0.523 \| - \| - \| - \| 0.908 \| - \| - \| 0.652 \| 0.908 \| \| 23 \| Osama 2023 \| Internal \| - \| 0.820 \| - \| - \| - \| 0.580 \| - \| - \| - \| 0.580 \| \| 24 \| Han 2023 \| Internal \| - \| 0.928 \| - \| - \| - \| 0.896 \| - \| - \| 0.909 \| 0.896 \| \| 25 \| Kaba 2023 \| Internal \| 0.900 \| 0.770 \| 0.983 \| 0.956 \| 0.869 \| 0.851 \| - \| - \| - \| - \| \| 26 \| Ling 2023 \| Internal \| 0.863 \| - \| - \| - \| - \| - \| - \|  \| - \| - \| \| 27 \| Yue 2024 \| Internal \| - \| 0.944 \| - \| 0.960 \| - \| 0.952 \| - \| - \| - \| - \| \| 28 \| Kim 2024 \| External \| - \| - \| - \| - \| - \| - \| - \| - \| 0.725 \| - \| \| 29 \| Labreque Langlais 2023 \| Internal \| - \| 0.729 \| 0.767 \| 0.394 \| - \| 0.512 \| - \| 20.2 \| 0.550 \| 0.829 \| \|  \|  \| External \| - \| 0.787 \| 0.928 \| - \| - \| - \|  \| 19.1 \| 0.679 \| 0.870 \| \| 30 \| In Kim 2024 \| Internal \| - \| 0.890 \| - \| - \| - \| - \| - \| - \| 0.833 \| - \| \| 31 \| Jimenez-Partinen 2024 \| Internal \| - \| - \| - \| - \| - \| 0.927 \| - \| - \| - \| 0.981 \| \| 32 \| Wang 2024 \| Internal \| 0.889 \| 0.854 \| - \| 0.875 \| - \| 0.871 \| - \| - \| - \| - \| \| 33 \| Begum 2024 \| Internal \| - \| 0.990 \| - \| 0.990 \| - \| 0.980 \| - \| - \| - \| - \| \| 34 \| Rostami 2024 \| Internal \| - \| 0.662 \| - \| 0.711 \| - \| 0.688 \| - \| - \| - \| - \| \| 35 \| Duan 2024 \| Internal \| 0.932 \| 0.935 \| - \| 0.929 \| - \| 0.932 \| - \| - \| - \| - \| \| 36 \| Chae 2025 \| External \| - \| 0.930 \| - \|  \| - \| - \| - \| - \| 0.725 \| - \| \| 37 \| Eschen 2025 \| Internal \| - \| 0.681 \| 0.922 \| 0.599 \| - \| 0.637 \| 0.178 \| - \| 0.661 \| 0.903 \| \|  \|  \| External \| - \| 0.548 \| 0.901 \| 0.220 \| - \| 0.314 \| 0.186 \| - \| 0.386 \| 0.833 \| |
| Moon 2023 and Kim 2025 excluded from this table as they did clinical validation studies instead of model development.  AUC: area under the ROC curve, MAE: mean absolute error, NPV: negative predictive value, PCC: Pearson correlation coefficien |

| Supplementary Table 7. Characteristics of included invasive physiological indices prediction studies |
| --- |
| \| No. \| First author \| Study type \| Input type \| Algoritme type \| Vessles \| Architecture \| Ground truth \| Training dataset \| Test dataset \| ICA indication \| Dataset origin \| Availability \| \| --- \| --- \| --- \| --- \| --- \| --- \| --- \| --- \| --- \| --- \| --- \| --- \| --- \| \| 1 \| Roguin 2020 \| External validation \| Multi frame \| Numerical prediction and binary classification \| LAD, LCx, RCA \| CNN \| FFR \| No training \| 31 patients \| Known or suspected CAD \| Clinical \| - \| \| 2 \| Ben-Assa 2023 \| External validation \| Multi frame \| Numerical prediction and binary classification \| LAD, LCx, RCA \| CNN \| FFR \| No Training* \| 297 patients \| CCS, UAP and NSTEMI \| Clinical \| - \| \| 3 \| Nobre-Menezes 2024 \| Model development \| Single frame + segmented frame \| Binary classification \| LAD, LCx, RCA \| CNN \| iFR \| 225 images \| 25 images \| CCS and ACS \| Clinical \| - \| \| 4 \| De Filippo 2024 \| Model development \| Multi frame \| Numerical prediction and binary classification \| WCT \| CNN + attention \| iFR and FFR \| 311 patients \| 78 patients \| CCS and ACS \| Clinical \| - \| \| 5 \| Arefinia 2024 \| Model development \| Single frame \| Binary classification \| LAD \| CNN \| FFR \| 2390 images \| 772 images \| Suspected CAD \| Clinical \| Model \| \| 6 \| Zhang 2024 \| Model development, external validation \| Multi frame \| FFR pullback curve prediction \| WCT \| Transformer-based \| Simulated FFR curves from CFD (training),  Distal invasive FFR (testing) \| 10634 images \| 544 images \| - \| Synthetic (training), clinical (testing) \| - \| \| 7 \| Mineo 2024 \| Model development \| Single frame \| Numerical prediction and binary classification \| WCT \| CNN-transformer hybrid \| iFR and FFR \| 622 images \| 156 images \| ACS and CCS \| Clinical \| Model \| \| 8 \| Oliveira 2025 \| Model development \| Single frame \| Binary classification \| LAD, LCx, RCA \| CNN \| iFR \| 200 images \| 50 images \| - \| clinical \| - \| \| 9 \| Omori 2025 \| External validation \| Multi frame \| Numerical prediction and binary classification \| LAD, LCx, RCA \| - \| Simulated iFR data based on CFD (training), iFR (testing \| 27000 images \| 178 patients \| - \| Synthetic (training), clinical (testing) \| - \| \| 10 \| Zhao 2023 \| Model development \| Multi frame \| CFR prediction \| WCT \| CNN \| Frame count based manual computation \| 4730 patients \| 1183 patients \| - \| Clinical \| - \| \| 11 \| Liu 2023 \| New application of existing model \| Multi frame \| CTO collateral physiology assessment \| WCT \| CNN \| Manual annotation \| No training \| 105 patients \| CTO PCI \| Clinical \| - \| |
| * Training details were not described; the model was developed by a commercial party. It was mentioned that training was performed using data from approximately 1,500 patients.  CFD: computational fluid dynamics, CNN: convolutional neural network, FFR: fractional flow reserve, ICA: invasive coronary angiography, iFR: instantaneous wave-free ratio, LAD: left anterior descending artery, LCx: left circumflex artery, RCA: right coronary artery, WCT: whole coronary tree. |

| Supplementary Table 8. Performance metrics for invasive physiological indices prediction models | |
| --- | --- |
| \| No. \| FIRST AUTHOR \| Dataset \| Accuracy \| Sensitivity \| Specificity \| precision \| NPV \| F1 \| MAE \| PCC \| AUC \| \| --- \| --- \| --- \| --- \| --- \| --- \| --- \| --- \| --- \| --- \| --- \| --- \| \| 1 \| Roguin 2020 \| External \| 0.900 \| 0.880 \| 0.930 \| 0.940 \| 0.870 \| - \| 0.050 \| 0.710 \| 0.910 \| \| 2 \| Ben-Assa 2023 \| External \| 0.837 \| 0.913 \| 0.945 \| 0.829 \| 0.974 \| - \| 0.005 \| 0.530 \| 0.930 \| \| 3 \| Nobre-Menezes 2024 \| Internal \| 0.690 \| 0.740 \| 0.670 \| 0.440 \| 0.880 \| - \| - \| - \| - \| \| 4 \| De Filippo 2024 \| Internal \| 0.873 \| 0.824 \| 0.922 \| 0.728 \| 0.954 \| - \| 0.037 (FFR)  0.026 (iFR) \| - \| 0.930 \| \| 5 \| Arefinia 2024 \| Internal \| 0.810 \| 0.860 \| 0.750 \| 0.820 \| - \| 0.840 \| - \| - \| 0.800 \| \| 6 \| Zhang 2024 \| Internal \| 0.922 \| - \| - \| - \| - \| - \| 0.023 \| 0.905 \| 0.938 \| \|  \|  \| External \| 0.930 \| - \| - \| - \| - \| - \| - \| 0.902 \| 0.912 \| \| 7 \| Mineo 2024 \| Internal \| 0.894 \| 0.800 \| 0.989 \| - \| - \| - \| 0.036 (FFR)  0.045 (iFR) \| - \| 0.950 \| \| 8 \| Oliveira 2025 \| Internal \| 0.720 \| 0.770 \| 0.710 \| 0.480 \| 0.900 \| - \| - \| - \| - \| \| 9 \| Omori 2025 \| External \| 0.766 \| 0.662 \| 0.815 \| 0.627 \| 0.837 \| - \| - \| 0.710 \| 0.810 \| \| 10 \| Zhao 2023 \| Internal \| - \| - \| - \| - \| - \| - \| - \| 0.510 \| - \| \| 11 \| Liu 2023 \| External \| - \| - \| - \| - \| - \| - \| - \| - \| - \| | |
| AUC: area under the ROC curve, MAE: mean absolute error, NPV: negative predictive value, PCC: Pearson correlation coefficient. | |
| Supplementary Table 9. Characteristics of included coronary anatomy labeling studies |  |
| \| No. \| First author \| Study type \| Input type \| Algorithm task \| Architecture \| Ground truth \| Training dataset \| Test dataset \| ICA indication \| Dataset origin \| Availability \| \| --- \| --- \| --- \| --- \| --- \| --- \| --- \| --- \| --- \| --- \| --- \| --- \| \| 1 \| Zhao 2023 \| Model development \| Single frame \| Semantic labeling \| GNN \| Manual \| 147 images \| 37 images \| - \| Clinical \| - \| \| 2 \| Cobo 2023 \| Model development \| Single frame \| Tortuosity detection \| CNN \| Manual \| 610 images \| 48 images \| Chest pain \| Clinical \| Model \| \| 3 \| Hatfaludi 2023 \| Model development \| Multi frame \| Collateral circulation detection \| CNN \| Manual \| 269 sequences \| 67 sequences \| - \| Clinical \| - \| \| 4 \| Moalla 2023 \| Model development \| Single frame \| LCA/RCA classification \| CNN \| Manual \| 8400 images \| 2100 images \| - \| Clinical \| Model, dataset partial \| \| 5 \| Zhao 2025 \| Model development, external validation \| Single frame \| Semantic labeling \| GNN \| Manual \| 410 images \| 45 images (internal)  263 images (external) \| - \| Clinical \| Model \| |  |
| CNN: convolutional neural network, GNN: graph neural network, ICA: invasive coronary angiography, LCA: left coronary artery, RCA: right coronary artery. |  |
| Supplementary Table 10. Performance metrics for coronary anatomy labeling models |  |
| \| No. \| First author \| Dataset \| Accuracy \| Sensitivity \| Precision \| Specificty \| F1-score \| AUC \| \| --- \| --- \| --- \| --- \| --- \| --- \| --- \| --- \| --- \| \| 1 \| Zhao 2023 \| Internal \| 0.865 \| 0.865 \| 0.866 \| - \| 0.864 \| - \| \| 2 \| Cobo 2023 \| Internal \| 0.870 \| 0.870 \| 0.890 \| 0.880 \| 0.870 \| 0.96 \| \| 3 \| Hatfaludi 2023 \| Internal \| 0.795 \| 0.804 \| - \| 0.786 \| - \| - \| \| 4 \| Moalla 2023 \| Internal \| 0.997 \| 0.961 \| 0.969 \| - \| 0.969 \| - \| \| 5 \| Zhao 2025 \| Internal \| 0.921 \| 0.926 \| 0.926 \| - \| 0.927 \| - \| \|  \|  \| External-1 \| 0.833 \| 0.840 \| 0.840 \| - \| 0.840 \| - \| \|  \|  \| External-2 \| 0.872 \| 0.879 \| 0.881 \| - \| 0.880 \| - \| |  |
| AUC: Area under ROC -curve |  |

| Supplementary Table 11. Characteristics of included reconstruction and registration studies. |
| --- |
| \| No. \| First author \| Study type \| Input type \| Algorithm task \| Architecture \| Ground truth \| Training dataset \| Test dataset \| ICA indication \| Dataset origin \| Availability \| \| --- \| --- \| --- \| --- \| --- \| --- \| --- \| --- \| --- \| --- \| --- \| --- \| \| 1 \| Fan 2019 \| Model development \| Image pairs \| Image registration \| CNN \| Manual labeling \| 40 images, 4000 correspondence points \| 10 images, 1000 correspondence points \| - \| Clinical \| - \| \| 2 \| Royer 2020 \| Model development \| Sequence pairs \| Cardiac cycle alignment \| CNN \| ECG \| 241 sequences \| 30 sequences \| - \| Clinical \| - \| \| 3 \| Fang 2020 \| Model development \| Multi frame \| respiratory signal estimation \| RNN+CNN \| Manual diaphragm tracing \| 243 sequences \| 173 sequences \| - \| Clinical \| - \| \| 4 \| Dinescu 2023 \| Model development \| Single frame \| Image quality enhancement \| CNN \| - \| 15000 images \| 4000 images \| - \| Clinical \| - \| \| 5 \| Bransby 2023 \| Model development \| Multi frame \| 3D reconstruction \| CNN+GNN \| Manual reconstruction \| 352 patients \| 62 patients \| CCS \| Clinical \| - \| \| 6 \| Yan 2023 \| Model development \| Single frame ICA + CCTA 3D centerline \| ICA to CCTA registration \| CNN+RL \| Mean projection error \| - \| - \| - \| Clinical \| - \| \| 7 \| HW Kim 2024 \| Model development + external validation \| Image pairs \| Image registration \| CNN + GNN \| Manual annotation \| 4781 image pairs \| 200 image pairs (internal)  56 image pairs (external) \| - \| Clinical, public \| Model, dataset partial \| \| 8 \| C Kim 2024 \| Model development \| Multi frame \| Image registration \| CNN \| Virtual guidewire \| 34 patients \| 51 patients \| - \| Clinical \| - \| \| 9 \| Li 2025 \| Model development + external validation \| OCT pullback + 2 ICA frames \| 3D fusion of ICA and OCT images \| Transformer \| Manual alginment \| 250 patients \| 28 patients (internal)  67 patients (OCT, ICA and CCTA) \| - \| Clinical \| - \| |
| CCTA: coronary computed tomography angiography, CCS: chronic coronary syndrome, CNN: convolutional neural network, GNN: graph neural network, ICA: invasive coronary angiography, OCT: optical coherence tomography, RL: reinforcement learning, RNN: recurrent neural network. |
| Supplementary Table 12. Performance metrics for reconstruction and registration models |
| \| No. \| First author \| Dataset \| Accuracy \| F1-score \| DSC \| IOU \| MAE (mm) \| HD (mm) \| MPE \| RMSE \| PCC \| \| --- \| --- \| --- \| --- \| --- \| --- \| --- \| --- \| --- \| --- \| --- \| --- \| \| 1 \| Fan 2019 \| Internal \| - \| - \| - \| - \| - \| - \| - \| 9.79 \| - \| \| 2 \| Royer 2020 \| Internal \| 0.960 \| - \| - \| - \| - \| - \| - \| - \| - \| \| 3 \| Fang 2020 \| Internal \| - \| - \| - \| - \| - \| - \| - \| 0.190* \| 0.790* \| \| 4 \| Dinescu 2023 \| Internal \| - \| - \| - \| - \| - \| - \| - \| - \| - \| \| 5 \| Bransby 2023 \| Internal \| - \| 0.826 \| 0.976 \| 0.786 \| 0.346 \| 1.188 \| - \| - \| - \| \| 6 \| Yan 2023 \| Internal \| - \| - \| - \| - \| - \| - \| 2.57 \| - \| - \| \| 7 \| HW Kim 2024 \| Internal \| 0.730 \| - \| - \| - \| - \| - \| - \| - \| - \| \|  \|  \| External \| 0.732 \| - \| - \| - \| - \| - \| - \| - \| - \| \| 8 \| C Kim 2024 \| Internal \| - \| - \| - \| - \| 1.03 \| - \| - \| - \| - \| \| 9 \| Li 2025 \| Internal \| - \| - \| - \| - \| 0.990 \| - \| - \| - \| - \| \|  \|  \| External \| - \| - \| - \| - \| - \| - \| - \| - \| - \| |
| * Metrics calculated on ICA images without diaphragm visible  DSC: Dice similarity coefficient, HD: Hausdorff distance, IOU: intersection over union, MAE: mean absolute error, MPE: mean projection error, PCC: Pearson correlation coefficient, RMSE: root mean square error. |

| Supplementary Table 13: Characteristics of included outcome prediction studies |
| --- |
| \| No. \| First author \| Study type \| Input type \| Algorithm task \| Architecture \| Ground truth \| Training dataset \| Test dataset \| ICA indication \| Dataset origin \| Availability \| \| --- \| --- \| --- \| --- \| --- \| --- \| --- \| --- \| --- \| --- \| --- \| --- \| \| 1 \| Thanou 2021 \| Model development \| Patch \| Predict MI culprit <5 years \| CNN \| EHR data \| 671 patches \| 75 patches \| - \| Clinical \| - \| \| 2 \| Perez-Martinez 2023 \| Model development \| Multi frame + clinical data \| Predict CTO procedural success \| CNN \| EHR data \| 90 patients \| 32 patients \| CTO PCI \| Clinical \| - \| \| 3 \| Mahendiran 2023 \| Model development \| Patch \| Predict MI culprit <5 years \| CNN \| EHR data, QFR if available \| 160 patches \| 40 patches \| NSTEMI, STEMI \| Trial (Future culprit) \| - \| \| 4 \| Sun 2024 \| Model development \| Multi frame + clinical data \| Cardiac event* prediction <2 years \| GNN \| EHR data \| 451 patients \| 112 patients \| CCS \| Trail (FAME2) \| Model \| \| 5 \| Sievering 2024 \| Model development \| Multi frame + clinical data \| Predict MI <5 years \| CNN-ANN combination \| Manual annotation + EHR data \| 356 patients \| 89 patients \| ACS \| Clinical \| - \| |
| *Cardiac death, MI, urgent and non-urgent revascularization  ACS: acute coronary syndrome, CCS: chronic coronary syndrome, CNN: convolutional neural network, CTO: chronic total occlusion, EHR: electronic health record, GNN: graph neural network, ICA: invasive coronary angiography, MI: myocardial infarction, NSTEMI: non–ST-elevation myocardial infarction, PCI: percutaneous coronary intervention, QFR: quantitative flow ratio, STEMI: ST-elevation myocardial infarction. |
| **Supplementary Table 14. Performance metrics for outcome prediction models** |
| \| No. \| FIRST AUTHOR \| Dataset \| accuracy \| Sensitivity \| Specificity \| precision \| NPV \| F1 \| AUC \| \| --- \| --- \| --- \| --- \| --- \| --- \| --- \| --- \| --- \| --- \| \| 1 \| Thanou 2021 \| Internal \| 0.775 \| 0.667 \| 0.806 \| - \| - \| 0.571 \| - \| \| 2 \| Perez-Martinez 2023 \| Internal \| 0.818 \| 0.875 \| 0.667 \| 0.875 \| 0.667 \| 0.875 \| 0.646 \| \| 3 \| Mahendiran 2023 \| Internal \| 0.780 \| 0.670 \| 0.810 \| 0.500 \| 0.890 \| - \| 0.810 \| \| 4 \| Sun 2024 \| Internal \| 0.720 \| 0.690 \| 0.730 \| 0.510 \| - \| 0.590 \| - \| \| 5 \| Sievering 2024 \| Internal \| - \| 0.600 \| 0.654 \| 0.100 \| - \| 0.167 \| 0.627 \| |
| AUC: area under the ROC curve, NPV: negative predictive value |

| Supplementary Table 15. Characteristics of LV function prediction study |
| --- |
| \| No. \| First author \| Study type \| Input type \| Algorithm task \| Architecture \| Ground truth \| Training dataset \| Test dataset \| ICA indication \| Dataset origin \| Availability \| \| --- \| --- \| --- \| --- \| --- \| --- \| --- \| --- \| --- \| --- \| --- \| --- \| \| 1 \| Avram 2023 \| Model development + external validation \| Multi frame \| LVEF prediction (binary class and numerical) \| CNN \| TTE derived LVEF \| 2727 patients \| 677 patients (internal)  744 patients (external) \| ACS and CCS \| Clinical \| - \| |
| ACS: acute coronary syndrome, CCS: chronic coronary syndrome, CNN: convolutional neural network, ICA: invasive coronary angiography, LVEF: left ventricular ejection fraction, TTE: transthoracic echocardiography. |
| Supplementary Table 16. Performance metrics of LV function prediction algorithm |
| \| No. \| First author \| Dataset \| Sensitivity \| Precision \| Specificty \| NPV \| AUC \| PCC \| ICC \| \| --- \| --- \| --- \| --- \| --- \| --- \| --- \| --- \| --- \| --- \| \| 1 \| Avram 2023 \| Internal \| 0.839 \| 0.490 \| 0.813 \| 0.960 \| 0.911 \| 0.710 \| 0.770 \| \|  \|  \| External \| 0.779 \| 0.660 \| 0.886 \| 0.934 \| 0.906 \| 0.670 \| 0.620 \| |
| NPV: negative predictive value, PCC: Pearson correlation coeficeint, AUC: , ICC |
